# Diagnostic performance of plasma Aβ42/40 ratio, p‐tau181, GFAP, and NfL along the continuum of Alzheimer's disease and non‐AD dementias: An international multi‐center study

**DOI:** 10.1002/alz.14573

**Published:** 2025-06-23

**Authors:** James D. Doecke, Giovanni Bellomo, Lisa Vermunt, Daniel Alcolea, Steffen Halbgebauer, Sjors in ’t Veld, Niklas Mattsson‐Carlgren, Katerina Veverova, Christopher J. Fowler, Lynn Boonkamp, Isabel M. Houtkamp, Marleen Koel‐Simmerlink, Inge M. W. Verberk, Lorenzo Gaetani, Andrea Toja, Anna Lidia Wojdała, Juan Fortea, Yolande Pijnenburg, Afina Lemstra, Wiesje van der Flier, Jakub Hort, Markus Otto, Oskar Hansson, Lucilla Parnetti, Colin L. Masters, Alberto Lleó, Armand González‐Escalante, José Contador, Marc Suárez‐Calvet, Aida Fernández‐Lebrero, Albert Puig‐Pijoan, Paula Ortiz‐Romero, Esther Jiménez‐Moyano, Carolina Minguillón, Marta del Campo, Charlotte Teunissen

**Affiliations:** ^1^ Australian E‐Health Research Centre CSIRO Herston Queensland Australia; ^2^ Section of Neurology Laboratory of Clinical Neurochemistry Department of Medicine and Surgery University of Perugia Perugia Italy; ^3^ Neurochemistry Laboratory Department of Laboratory Medicine Amsterdam Neuroscience Amsterdam University Medical Center Vrije Universiteit Amsterdam the Netherlands; ^4^ Alzheimer Center Amsterdam Department of Neurology Amsterdam Neuroscience Amsterdam University Medical Centers Amsterdam the Netherlands; ^5^ Department of Neurology Institut d'Investigacions Biomèdiques Sant Pau ‐ Hospital de Sant Pau Universitat Autònoma de Barcelona Hospital de la Santa Creu i Sant Pau Barcelona Spain; ^6^ Department of Neurology University of Ulm Ulm Germany; ^7^ Translational AI in Laboratory Medicine Department of Laboratory Medicine Location VUmc Amsterdam the Netherlands; ^8^ Clinical Memory Research Unit Department of Clinical Sciences Malmö Faculty of Medicine Lund University Lund Sweden; ^9^ Neurology Clinic Skåne University Hospital Malmö Sweden; ^10^ Wallenberg Center for Molecular Medicine Lund University Lund Sweden; ^11^ Memory Clinic Department of Neurology Charles University Second Faculty of Medicine and Motol University Hospital Prague Czech Republic; ^12^ The University of Melbourne The Florey Institute Victoria Melbourne Australia; ^13^ Memory Clinic Skåne University Hospital Malmö Sweden; ^14^ Barcelonaβeta Brain Research Center (BBRC) Pasqual Maragall Foundation Sant Martí Barcelona Spain; ^15^ Department of Neurology Hospital del Mar Research Institute Ciutat Vella Barcelona Spain; ^16^ Faculty of Medicine and Life Sciences Universitat Pompeu Fabra Carrer de la Mercè Ciutat Vella Barcelona Spain; ^17^ Cognitive and Behavioural Neurology Unit Department of Neurology Hospital del Mar Ciutat Vella Barcelona Spain; ^18^ Centro de Investigación Biomédica en Red de Fragilidad y Envejecimiento Saludable (CIBERFES) Instituto de Salud Carlos III Madrid Spain; ^19^ Departamento de Ciencias Farmacéuticas y de la Salud Facultad de Farmacia Universidad San Pablo‐CEU CEU Universities, Urbanización Montepríncipe Fuencarral‐El Pardo Spain

**Keywords:** Alzheimer's disease, amyloid beta, dementia with Lewy bodies, frontotemporal dementia, plasma biomarkers

## Abstract

**INTRODUCTION:**

Plasma phosphorylated tau (p‐tau)181, glial fibrillary acidic protein (GFAP), neurofilament light chain (NfL), and amyloid beta ratio (Aβ42/40) may have diagnostic and prognostic value in Alzheimer's disease (AD). Here we assess which markers can best identify AD from controls and other non‐AD dementias in a large international multi‐center study.

**METHODS:**

Plasma samples (*n* = 1298) were collected from six international centers. Aβ40, Aβ42, GFAP, NfL, and p‐tau181 were measured using single molecule array. In each group, AD diagnosis/co‐pathology was defined according to cerebrospinal fluid biomarkers or amyloid positron emission tomography. Validations were performed in three separate cohorts via single and dual cut‐off models.

**RESULTS:**

p‐tau181 showed the best area under the curve value to separate AD from frontotemporal dementia, controls, and Aβ– dementia with Lewy bodies. However, this discriminative power could not be reproduced by applying pre‐defined cut‐offs.

**DISCUSSION:**

p‐tau181 was the best single plasma marker for detecting AD at any stage. Specific cut‐offs are needed to maximize diagnostic performances.

**Highlights:**

- Phosphorylated tau (p‐tau)181 provided a clear differentiation between controls and Alzheimer's disease (AD) participants, with evidence of increased levels in the preclinical stage of AD.
- Plasma biomarkers demonstrated that when amyloid co‐pathology is removed from dementia with Lewy bodies (DLB), only glial fibrillary acidic protein and neurofilament light chain remain to predict DLB.
- Given the low prevalence of amyloid co‐pathology in frontotemporal dementia (FTD), p‐tau181 and its ratio with amyloid beta 42 are strong biomarkers to differentiate FTD from AD.

## 1 BACKGROUND

Accurate detection of Alzheimer's disease (AD) pathology to support clinical diagnosis has in the past been limited by the use of either amyloid beta (Aβ) positron emission tomography (PET) imaging or cerebrospinal fluid (CSF) biomarker analyses.^1^ The availability of these tools is generally low, with varying access in different countries and health‐care systems. The associated costs of CSF and/or PET imaging and cognitive assessments to the public health‐care system together with the burden to the patient and their families from such testing are high.^2^ Furthermore, Aβ pathology can be present in 50% of patients with dementia with Lewy bodies (DLB) and in 25% of patients with frontotemporal dementia (FTD) syndromes,^3^ thus highlighting the need to have specific instruments to discriminate across diseases. In DLB, an accurate diagnosis requires an intensive psychological assessment, with biomarkers from single‐photon emission computed tomography, PET, or ^123^iodine‐metaiodobenzylguanadine myocardial scintigraph not required but used as positive indicators.^4^ For the detection of FTD pathology, expensive imaging ([18F]‐fluorodeoxyglucose [FDG] PET, diffusion tensor imaging [DTI], resting‐state functional magnetic resonance imaging, tau PET), and/or genetic testing are needed.^5^, ^6^

Recent scientific advances enabled the measurement of Aβ42/40 ratio and different forms of phosphorylated tau (p‐tau) 181, 205, 217, or 231 in plasma, as biomarkers of Aβ plaques and neurofibrillary tangles. Other relevant mechanisms that are not specific for AD such as astrogliosis and neurodegeneration can also be detected using plasma glial fibrillary acidic protein (GFAP), and neurofilament light (NfL), respectively.^7^, ^8^ The markers combined allow a better molecular characterization of neurodegenerative diseases, thereby supporting better design of appropriate treatment strategies.

For AD, the amyloid/tau/neurodegeneration or “AT(N)” criteria, using CSF and/or PET imaging, has been instrumental in accurately detecting both the presence of Aβ and tau pathology, and to stage patients along the AD continuum.^9^ An update to the criteria^10^ recently has introduced high accuracy plasma biomarkers being interchangeable with CSF biomarkers for the positive identification of AD pathology. Single‐center biomarker studies including different dementia types showed that Aβ42/40 was decreased only in those with Aβ pathology; p‐tau181 was increased mainly in those with AD, but increases during preclinical AD (preAD) and mild cognitive impairment (MCI) prior to clinical AD dementia; GFAP, defined as a marker of Aβ‐related astrocyte reactivity in autosomal dominant AD^11^ was increased in all dementias compared to controls;^12^, ^13^ and NfL had the highest values/concentrations in patients with FTD. p‐tau181, p‐tau231,^14^ and Aβ42/40,^15^ but not GFAP or NfL, have been shown to be increased in DLB compared to controls (however, this was only seen in those with Aβ co‐pathology). No other information was shown to determine the performance of these markers to discriminate AD from other non‐AD dementias along their symptomatic continuum (e.g., MCI due to FTD, or FTD).

In the present study, we evaluate the performance of the most frequently used plasma biomarker assays to detect Aβ pathology in a large international multi‐center cohort. We assess the levels of Aβ42/40, p‐tau181, p‐tau181/Aβ42, GFAP, and NfL across the AD, FTD, and DLB disease continuums, including the effect of Aβ co‐pathology in DLB. Next, we define cut‐offs to predict the likelihood of clinical stage within AD, which we then tested in multiple validation cohorts.

**RESEARCH IN CONTEXT**

1. **Systematic review**: A comprehensive search of the PubMed literature was conducted to identify published studies which assessed plasma biomarkers and their association with neurological disease. Recent publications have shown mixed results in predicting amyloid beta postivity or disease stage using plasma biomarkers. To address this directly, the present study showcases results from a large international cohort from six separate studies with central assessment of key plasma biomarkers to detect disease stage across the Alzheimer's disease (AD), dementia with Lewy bodies (DLB), and frontotemporal dementia (FTD) disease continuums, and with validation in three independent cohorts with independent plasma biomarker assessment.
2. **Interpretation**: Our findings demonstrate that using a large international cohort was able to discern disease stage across the AD, DLB, and FTD continuums. For DLB, biomarker value is dependent on the presence of amyloid co‐pathology. For FTD, AD‐specific biomarkers provide no further diagnostic ability from controls over neurofilament light chain, while they are of use for differential diagnosis from AD.
3. **Future directions**: Further work is needed to identify plasma biomarkers, or panels of plasma biomarkers, for both the diagnosis of DLB and FTD, and the differential diagnosis between AD, DLB, and FTD.

## 2 METHODS

### 2.1 Participants

Samples were collected across six different international centers: Amsterdam Dementia Cohort (ADC; all groups^16^), Sant Pau Initiative on Neurodegeneration (SPIN; all groups^13^), University of Perugia (UNIPG; all groups^17^), Ulm University (UU; all groups except MCI‐DLB^18^), BioFINDER (controls and complete AD continuum^19^), and the Australian Imaging Biomarker & Lifestyle Flagship Study of Ageing (AIBL; controls and complete AD continuum^20^) as part of the blood proteins for early discrimintation of dementia's (bPRIDE) study. Three additional independent cohorts were used to validate the resulting cut‐offs across the AD continuum: independent samples from UNIPG^21^ (22 controls, 59 along the full AD continuum, and 17 FTD^22^), ALzheimer's and FAmilies (ALFA+; 265 controls [CSF Aβ–] and 135 preAD [CSF Aβ+]^23^, ^24^), and BIODEGMAR (individuals who visited the memory clinic with AD CSF profile [*n* = 112] or with non‐AD CSF profile [*n* = 61]^25^). For all the samples, the presence of AD pathology was tested either by using AD CSF biomarkers (CSF Aβ_42_ or Aβ_42/40_, total tau [t‐tau], and p‐tau181, “AD CSF biomarkers”; all cohorts) or amyloid and tau PET (most samples from the AIBL cohort).

The total cohort (*n* = 1298) of this cross‐sectional study included plasma samples from controls without (biomarker signs of) Aβ pathology (*n* = 198); controls with Aβ pathology (preAD; *n* = 155), patients with stable MCI without Aβ pathology (sMCI, *n* = 170), patients with Aβ pathology and MCI due to AD (MCI[AD]; *n* = 155), MCI due to FTD (MCI[FTD]; *n* = 46), MCI due to DLB (MCI[DLB]; *n* = 25), AD dementia (*n* = 182), FTD (*n* = 170), and DLB (*n* = 171). In the DLB group, 99 of the 171 patients were Aβ positive, and 60 were Aβ negative (12 were missing Aβ status). The control group included individuals with subjective cognitive decline, in whom objective cognitive and laboratory investigations were normal (i.e., criteria for MCI, dementia, or any other neurological or psychiatric disorder not fulfilled) with additionally negative AD CSF biomarkers.^16^, ^26^ For the ease of labeling throughout this work, the abbreviation “controls” is used to reflect this group as a whole.

All participants of each cohort underwent standard neurological and cognitive assessments, and the diagnosis was assigned according to international consensus criteria for MCI(AD),^27^ AD dementia,^28^ DLB,^4^, ^29^, and FTD.^30^, ^31^, ^32^ DLB cases were further split for Aβ co‐pathology. Of the 171 participants with DLB, 12 did not have Aβ status information (Table S1 in supporting information). The MCI(DLB) group contained only 25 participants, and as such was not investigated other than plotting. Blood sampling has been performed in each of the centers following harmonized procedures.^33^ The main pre‐analytical difference across centers was the addition of prostaglandin E1 to ethylenediaminetetraacetic acid tubes in samples collected by AIBL. Mini‐Mental State Examination (MMSE) or Montreal Cognitive Assessment (MoCA) tests were used as a measure of global cognition. CSF markers were analyzed locally as part of the diagnostic procedure using commercially available kits (ADC, AIBL, and UU: enzyme‐linked immunosorbent assay [ELISA] INNOTEST, Fujirebio; ADC and ALFA+: Elecsys biomarker assays, Roche Diagnostics GmbH; SPIN, UNIPG, UU, and BIODEGMAR: Lumipulse G600, Fujirebio; BioFINDER: Euroimmune ELISA assays).^13^, ^34^ Positive CSF AD biomarker profile was defined locally in the different cohorts (ADC [Aβ+ Innotest based on Aβ_42_ < 813; Elecsys Aβ_42_ < 1000,^35^ Tau+ Innotest pTau > 52, Elecsys pTau > 24^34^], BioFINDER [CSF Aβ_42_/Aβ_40_ < 0.088, p‐tau181 > 65.04 pg/mL or t‐tau > 462.91 pg/mL]; SPIN [low Aβ_42/40_ ratio (< 0.062) and high t‐tau (> 456pg/mL) or p‐tau (> 63pg/mL)]; UNIPG [low Aβ_42/40_ ratio (< 0.072) and high p‐tau (> 50pg/mL), regardless of t‐tau]; UU [low Aβ_42_ (< 450pg/mL) and high t‐tau (> 380pg/mL) or p‐tau (> 65pg/mL)]; ALFA+ [Aβ_42_/Aβ_40_ < 0.071, p‐tau181 < 24pg/mL^23^]; BIODEGMAR [Aβ_42_/Aβ_40_ < 0.062^13^, ^36^, ^37^]). All samples from the AIBL cohort were defined based on Aβ and tau PET (amyloid PET positive: > 25 Centiloids,^38^ tau PET positive: meta‐temporal standardized uptake value ratio > 1.19^39^).

### 2.2 Plasma biomarker measurements

Samples from each center of the discovery cohort were all shipped for central measurement at the neurochemistry laboratory of the Amsterdam University Medical Center using the single‐molecule array (Simoa) technology in a Simoa HD‐X Analyzer;^40^, ^41^ Quanterix Corp.). Plasma p‐tau181 was quantified over 13 runs using the Simoa pTau‐181 Advantage v2 kit (kit #103714, lot #502923; Quanterix Corp.) in duplo with on‐board automated sample dilution, according to the manufacturer's instructions. Plasma Aβ40, Aβ42, GFAP, and NfL concentrations were quantified over six runs using the Simoa Neurology 4‐PLEX E (N4PE) Advantage kit (item# 103971, lot #503413; Quanterix Corp.) in monoplo with on‐board automated sample dilution, according to the manufacturer's instructions. The plasma measurements of the validation cohorts were measured locally using similar N4PE kits (UNIPG: kit: #103670, lot #503213; ALFA+ & BIODEGMAR: kit #103670, lot #503819) and p‐tau181 kits (UNIPG, Simoa pTau‐181 Advantage v2: kit #103714; ALFA+: Simoa pTau‐181 Advantage v2.1 kit #104111, lots #503680 & #503769; and BIODEGMAR: Simoa pTau‐181 Advantage v2.1: kit #104111, lot #503537). Plasma p‐tau181 values from ALFA+ and BIODEGMAR were thus corrected for the kit version employed following the formula provided by the manufacturer. No further harmonization in plasma levels to bPRIDE data was done for the validation cohorts.

### 2.3 Statistical methods

All data preprocessing and analyses were conducted using R version 4.2.2.^42^ Between‐group analyses for the demographic variables were performed using chi‐squared test for categorical variables and independent samples *t* test for quantitative variables. Possible center‐specific differences in the data were evaluated via principal component analyses (PCA, Figure S1 in supporting information). Mean plasma biomarker levels were compared between disease groups using the binary generalized linear model (GLM), both unadjusted and adjusted for age, sex, the first three components from the PCA analyses, and apolipoprotein E (*APOE*) ε4 allele status where appropriate. The ratio between p‐tau181 and Aβ42 was assessed and added to each analysis given previously seen increases in association with amyloid burden over p‐tau181 or Aβ42 alone.^43^ Multivariate analyses to identify the optimal combination of biomarkers per disease was performed by minimizing the Akaike information criterion (AIC) via stepwise assessment in GLMs if the sample size was not prohibitive.^44^ To investigate whether any combination of individual biomarkers could predict disease group, all possible ratios and interactions between biomarkers (biomarker ratios and biomarker × biomarker interactions) were tested using the least operating shrinkage and selection operator (LASSO) method.^44^

For receiver operating characteristic (ROC) analyses, plasma data were assessed against binary disease categories (Table S1) using a GLM. Area under the curve (AUC) values were calculated from rolling circle cross‐validation (referred to below as internal cross validation), where the complete set of samples, minus the complete set of samples from one center (hold out or test set), were modeled using a GLM as the training set. The resulting model coefficients were used to predict disease categories on the test set. This approach was repeated where each study site with enough samples (> *N* = 20 per group) was used as the test set. Final AUC values were calculated from the average result from each test set accounting for possible center differences in AUC. Cut‐off derivation using the dual cut‐off model (90% sensitivity, 90% specificity) along with Youden index was performed using the complete bPRIDE data set. The proportion of values in the gray zones were calculated in two ways: (1) using the proportion of participants whose plasma biomarker concentration falls within the gray zone divided by the total number of participants, and (2) the proportion of values in the gray zones were calculated by the frequency of values between the two thresholds divided by the total range of the data.

External cross‐validations were performed using two separate methods on three separate validation cohorts. Method 1 (separate to rolling circle cross‐validation described above) involved setting up the model on the training set (complete bPRIDE data set) and then validating model coefficients on the test data set (external data). Method 2 used biomarker cut‐offs derived from the complete bPRIDE data set (at Youden index) to derive binary biomarkers (low/high) within the validation cohort before running an AUC between the newly created binary biomarker and the outcome. For the first validation cohort (UNIPG), both validation methods were used. For the second (ALFA+) and third (BIODEGMAR) cohorts, only validation Method 2 was used, as the performance of the analyzed markers in these cohorts have been already published.^25^, ^45^ AUC with 95% confidence intervals (CIs), sensitivity, specificity, positive predictive value (PPV, not prevalence adjusted), negative predictive value (NPV, not prevalence adjusted), cut‐off (at Youden index, and at 90% sensitivity and specificity), and accuracy are reported.

## 3 RESULTS

### 3.1 Sample demographics

Of the complete bPRIDE sample set, 1298 samples with available plasma measurements were included in the analysis (Table 1). Across diagnostic groups on average, 48% were male, the mean age was 70.1 years (standard deviation [SD]: 7.1), and 32% carried at least one *APOE* ε4 allele (where measured). Subjects with preAD, MCI(AD), AD dementia, MCI(DLB), and DLB were older than controls and were more frequently *APOE* ε4 carriers compared to controls. All cases along the AD continuum (i.e., preAD, MCI[AD], and AD dementia) were Aβ+, while only a small group of those with MCI(FTD) or FTD were Aβ+ (11% and 8%, respectively). Approximately half of all participants with DLB (MCI/DLB) had Aβ co‐pathology. MMSE was lower in all groups compared to controls except for those with preAD. Across centers (Table S1), mean age ranged between 66.6 years of age (SD: 6.43, ADC) through 72.24 years of age (SD: 7.29, SPIN). Median MMSE across centers ranged between 25 (median absolute deviation [MAD]: 4.45, ADC, UNIPG, UU, and SPIN) and 29 (MAD: 1.48, BioFINDER). Median years of education across centers ranged between 8 years (SD: 4.45, UNIPG) and 12 (SD: 2.97–4.45, AIBL, UU, and BioFINDER).

**TABLE 1:** Study demographic parameters.

	Total sample	Controls	sMCI	preAD	MCI(AD)	AD dementia	MCI(FTD)	FTD	MCI(DLB)	DLB
*N* (%)	1298	198 (15%)	170 (13%)	181 (14%)	155 (12%)	182 (14%)	46 (4%)	170 (13%)	25 (2%)	171 (13%)
Sex male, *N* (%)	626 (48%)	84 (42%)	81 (48%)	61 (34%)	68 (44%)	70 (38%)	30 (65%)	97 (57%)	15 (60%)	120 (70%)
Mean age, years (SD)	70.1 (7.1)	68.1 (7.4)	69 (7.6)	71.9 (5.9)	71.9 (6)	70.6 (7.5)	68.5 (6.3)	67.9 (7.9)	70.8 (6)	72 (6.3)
*APOE* ɛ4 carriage, *N* (%)	418 (32%)	18 (9%)	32 (19%)	75 (41%)	89 (57%)	92 (51%)	4 (9%)	24 (14%)	7 (28%)	77 (45%)
Aβ+	516 (40%)	0 (0%)	6 (4%)	181 (100%)	155 (100%)	182 (100%)	5 (11%)	13 (8%)	12 (48%)	86 (50%)
Median MMSE, (MAD)	25 (4.7)	28.6 (1.5)	26.4 (2.4)	28.4 (1.4)	25.7 (2.6)	19.4 (5.4)	25.9 (2.7)	22.7 (5)	25.9 (3)	22.3 (4.5)

Abbreviations: Aβ, amyloid beta; AD, Alzheimer's disease; *APOE*, apolipoprotein E; DLB, dementia with Lewy bodies; FTD, frontotemporal dementia; MAD, median absolute deviation; MCI, mild cognitive impairment; MMSE, Mini‐Mental State Examination; *N*, number; preAD, pre‐clinical Alzheimer's disease; SD, standard deviation; sMCI, stable mild cognitive impairment.

### 3.2 Biomarker measurements across centers

**FIGURE 1:**
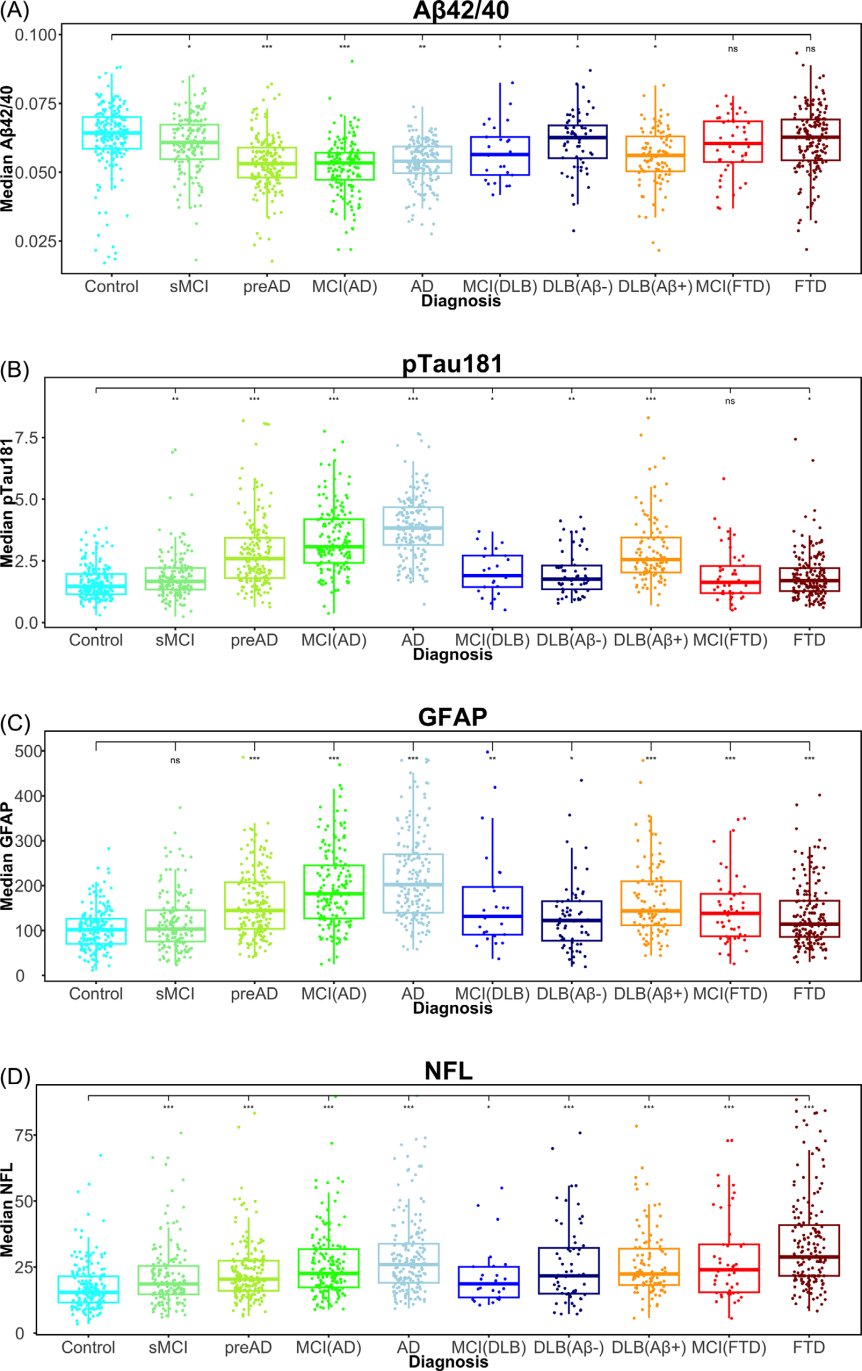
Box and whisker plots of plasma biomarkers over the disease continuums for AD, DLB, and FTD. (A) Aβ42/40, (B) p‐tau181, (C) GFAP, and (D) NfL. – *P* > 0.05, ^*^ *P* < 0.05, ^**^ *P* < 0.01, ^***^ *P* < 0.001, ^****^ *P* < 0.0001 versus controls. Values shown for Aβ42, GFAP, NfL, and p‐tau181 are in pg/mL. Aβ, amyloid beta; AD, Alzheimer's disease; DLB, dementia with Lewy bodies; FTD, frontotemporal dementia; GFAP, glial fibrillary acidic protein; MCI, mild cognitive impairment; NfL, neurofilament light chain; preAD, pre‐clinical AD; p‐tau, phosphorylated tau; sMCI, stable MCI

Figure S1C shows most of the variance across the biomarkers tested was explained within the first component (≈ 85%), with little to no variance seen across center (Figure 1A). To assess the relative contribution of center within ROC analyses, the first three principal components (PCs) were modeled along with each biomarker to predict AD from controls (we used this comparison as a representative to assess center differences only), with AUC values compared to using individual biomarkers to predict AD from controls. Modest to strong improvements were seen in AUC values when models included three PCs, with Aβ42/40 and NfL showing the largest increase in AUC values after accounting for PCs (*P* < 0.0001), with p‐tau181 having only a small increase in AUC value (*P* = 0.015) and GFAP having no increase (*P* = 0.22). Thus, overall performance was increased after accounting for center, except for GFAP, which remained the same (Table S2 in supporting information). Further testing for possible center differences showed that biomarker AUC values (predicting AD from controls) were not significantly different between centers (Table S2). AUC values presented from here are taken from the average of the rolling circle validation analyses, such that no center biases in AUC values are presented.

We will next report results along the disease continuum for each of the dementia types separately.

### 3.3 Biomarker combinations to detect amyloid positivity within the AD continuum

**FIGURE 2:**
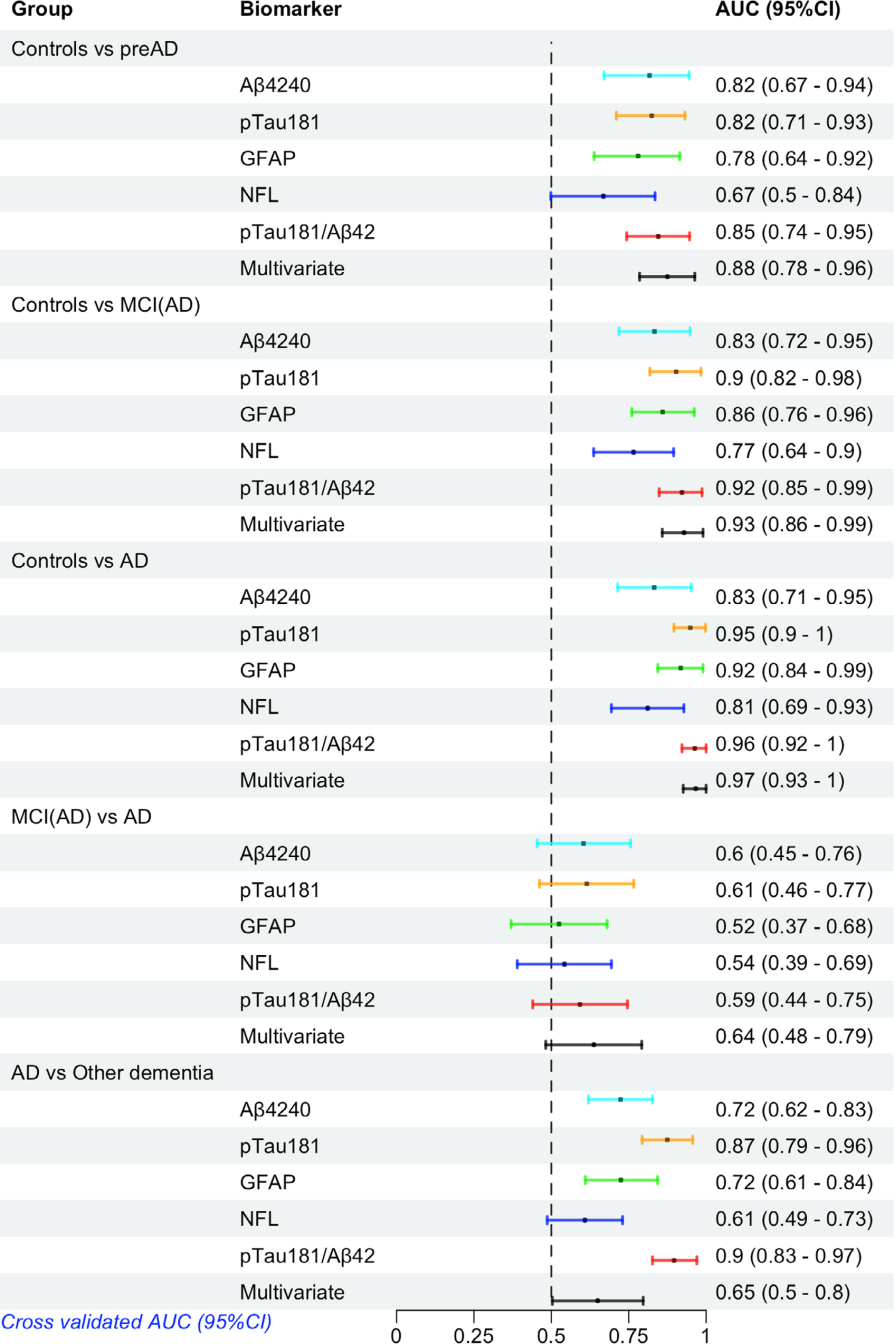
Internal cross‐validation AUC (95% CI) values for the AD continuum and for AD versus other dementia. Other dementia: includes participants with DLB or FTD. Multivariate model included the interaction Aβ40 × p‐tau181, and ratios Aβ42/GFAP and Aβ42/p‐tau181. AD, Alzheimer's disease; AUC, area under the curve; CI, confidence interval; DLB, dementia with Lewy bodies; FTD, frontotemporal dementia; GFAP, glial fibrillary acidic protein; MCI, mild cognitive impairment; NfL, neurofilament light chain; preAD, pre‐clinical AD; p‐tau, phosphorylated tau

Compared to the control group, all biomarkers analyzed were already changed in the preAD group and continued increasing (except for the Aβ42/40 ratio which decreased to its lowest level in preAD) through the MCI(AD) and dementia due to AD stages. Among the biomarkers, plasma p‐tau181 and GFAP showed the largest stepwise increased changes over the full AD continuum (Table 2, Figure 1A–D). We next evaluated which combination of markers (single, ratios, and interactions, into a multivariate model) had the best performance to discriminate any stage of the AD continuum compared to controls. The sMCI group without Aβ pathological changes had only low to moderate changes in all biomarker levels and given such small changes along with high variation, was not further considered in predictive modeling. Regarding single markers and Aβ42/40 and p‐tau181/Aβ42 ratios, p‐tau181 and the p‐tau181/Aβ42 ratio showed the highest discriminative performance across all disease stages compared to controls (AUC > 0.82), with no significant differences in AUC values among p‐tau181, p‐tau181/Aβ42, and Aβ42 (Figure 2). No biomarkers were able to efficiently discriminate AD dementia from MCI(AD), with all AUC values < 0.65. Plasma p‐tau181 and p‐tau181/Aβ42 showed the strongest performance (AUC 0.87 and 0.90, respectively) to discriminate AD from the group of non‐AD dementias (FTD Aβ– and DLB Aβ–).The multivariate model (chosen consistently across all group comparisons; ROC statistics shown in Table 3 compared to individual plasma biomarkers), comprising the pre‐calculated interaction between Aβ40 × p‐tau181, and ratios Aβ42/GFAP and Aβ42/p‐tau181, had significantly higher AUC values compared to the top performing individual biomarker; however, such differences in AUC diminished with increasing disease stage.

**TABLE 2:** Mean biomarker levels per clinical group.

Biomarker	Controls	sMCI	preAD	MCI(AD)	AD dementia	MCI(DLB)	DLB	MCI(FTD)	FTD
Aβ42	6.19 (1.64)	6.34 (1.61)	5.36 (1.44)**	5.34 (1.53)**	5.46 (1.28)**	6.2 (1.52)	6.15 (1.66)‘‘	6.2 (1.14)	6.33 (1.64) †††
Aβ42/40	0.063 (0.012)	0.06 (0.01)	0.053 (0.0099)***	0.052 (0.0098)***	0.054 (0.008)***	0.06 (0.011)	0.058 (0.011)*** ‘‘	0.060 (0.011)	0.062 (0.012)†††
GFAP	104 (45.9)	122 (63.5)*	160 (74.1)***	195 (87.3)***	219 (95.5)***	146 (77.4)*	154 (83.2)*** ‘‘‘	146 (77.4)***	133(69.7)*** ††† ^^^
NfL	17.5 (9.01)	21.4 (11.8)**	23 (11.1)***	26 (12.8)***	29 (14.7)***	28.9 (16.8)	26 (13.6)*** ‘	28.9 (16.8)***	33.9 (18)*** †† ^^^
p‐tau181	1.63 (0.66)	1.92 (0.978)*	2.83 (1.44)***	3.37 (1.34)***	3.85 (1.27)***	1.93 (1.09)	2.64 (1.34)*** ‘‘‘	1.93 (1.09)	1.88 (0.956)* ††† ^^^
p‐tau181/Aβ42	0.29 (0.181)	0.316 (0.175)	0.531 (0.275)***	0.694 (0.323)***	0.736 (0.278)***	0.361 (0.188)	0.46 (0.291)*** ‘‘‘	0.347 (0.249)	0.334 (0.249)††† ^^^

*Note*: Values shown for Aβ42, GFAP, NfL, and p‐tau181 are in pg/mL. Comparisons of each group back to controls: *< 0.05, **< 0.01, ^***^< 0.001. Comparison of AD dementia versus DLB: ^‘^< 0.05, ^‘‘^< 0.01, ^‘‘‘^< 0.001. Comparison of AD dementia versus FTD: ^†^< 0.05, ^††^< 0.01, ^†††^< 0.001. Comparison of DLB versus FTD: ^^^< 0.05, ^^^^< 0.01, ^^^^^< 0.001.

Abbreviations: Aβ, amyloid beta; AD, Alzheimer's disease; DLB, dementia with Lewy bodies; FTD, frontotemporal dementia; GFAP, glial fibrillary acidic protein; MCI, mild cognitive impairment; NfL, neurofilament light chain; preAD, pre‐clinical Alzheimer's disease; p‐tau, phosphorylated tau; sMCI, stable mild cognitive impairment.

**TABLE 3:** Multivariate analyses for the AD continuum.

	Controls versus preAD		Controls versus MCI(AD)		Controls versus AD dementia	
	p‐tau181	MV model	p‐tau181	MV model	p‐tau181	MV model
AUC (95% CI)	0.80 (0.75–0.84)	0.85 (0.81–0.89)	0.90 (0.86–0.93)	0.93 (0.9–0.96)	0.95 (0.92–0.97)	0.96 (0.95–0.98)
Sensitivity	68.51	71.27	79.35	89.03	84.07	88.46
Specificity	77.78	86.87	87.37	85.86	89.9	91.92
Cut‐off	2.01	0.552	2.31	0.402	2.49	0.489
PPV	73.81	83.23	83.11	83.13	88.44	90.96
NPV	72.99	76.79	84.39	90.91	85.99	89.66
Accuracy	73.35	79.42	83.85	87.25	87.11	90.26

*Note*: Multivariate models all included the interaction Aβ40 × p‐tau181, and ratios Aβ42/GFAP and Aβ42/p‐tau181. Cut‐off values are provided in pg/mL for Aβ42, GFAP, NfL, and p‐tau181.

Abbreviations: AD, Alzheimer's disease; AUC, area under the curve; CI, confidence interval; MCI, mild cognitive impairment; MV, multivariate; NPV, negative predictive value; PPV, positive predictive value; preAD, pre‐clinical Alzheimer's disease; p‐tau, phosphorylated tau.

### 3.4 Controls versus DLB continuum

**FIGURE 3:**
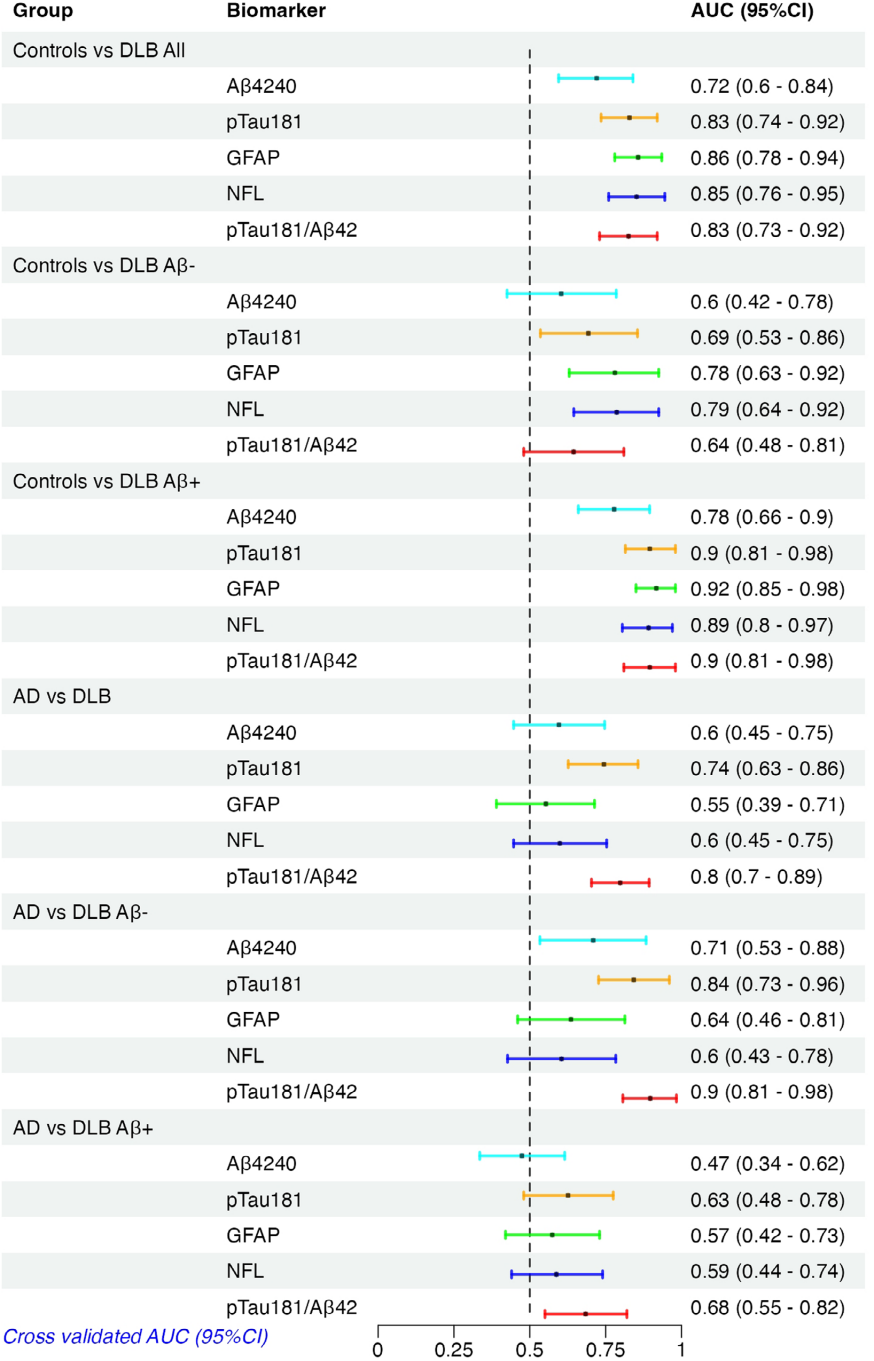
Internal cross‐validation AUC (95% CI) values for the DLB Aβ‐ and DLB Aβ+ versus controls, and between DLB & AD. Aβ, amyloid beta; AD, Alzheimer's disease; AUC, area under the curve; CI, confidence interval; DLB, dementia with Lewy bodies; GFAP, glial fibrillary acidic protein; NfL, neurofilament light chain; p‐tau, phosphorylated tau

Irrespective of Aβ co‐pathology and compared to the control group, all biomarkers (except for Aβ42) were increased in DLB (Aβ42/40 was decreased), while only GFAP was significantly increased in those with MCI(DLB) (Table 2). Considering the absence/presence of Aβ co‐pathology, Aβ42/40 was decreased and p‐tau181 and GFAP were only increased in the DLB Aβ+ group compared to controls, and not altered in the DLB Aβ– group (Figure 1). NfL, however, was increased in both DLB Aβ– and DLB Aβ+ groups compared to controls. Assessing the diagnostic capability of each biomarker irrespective of Aβ co‐pathology (Figure 3), GFAP had the highest AUC value (AUC: 0.86 [95% CI: 0.78–0.94]); however, this was not significantly different from all other markers except for Aβ42/40, which had the lowest AUC value (AUC: 0.72 [95% CI: 0.60–0.84]).

When Aβ pathology was present, biomarker AUC values for the comparison of DLB versus controls were similar to that for the AD continuum (all AUC > 0.78). When no Aβ pathology was present, only NfL and GFAP tended to be increased in DLB patients compared to controls (AUC > 0.78). With only 25 patients with MCI(DLB), this could not be validated during the earlier stage of the DLB continuum.

### 3.5 Controls versus frontotemporal dementia continuum

**FIGURE 4:**
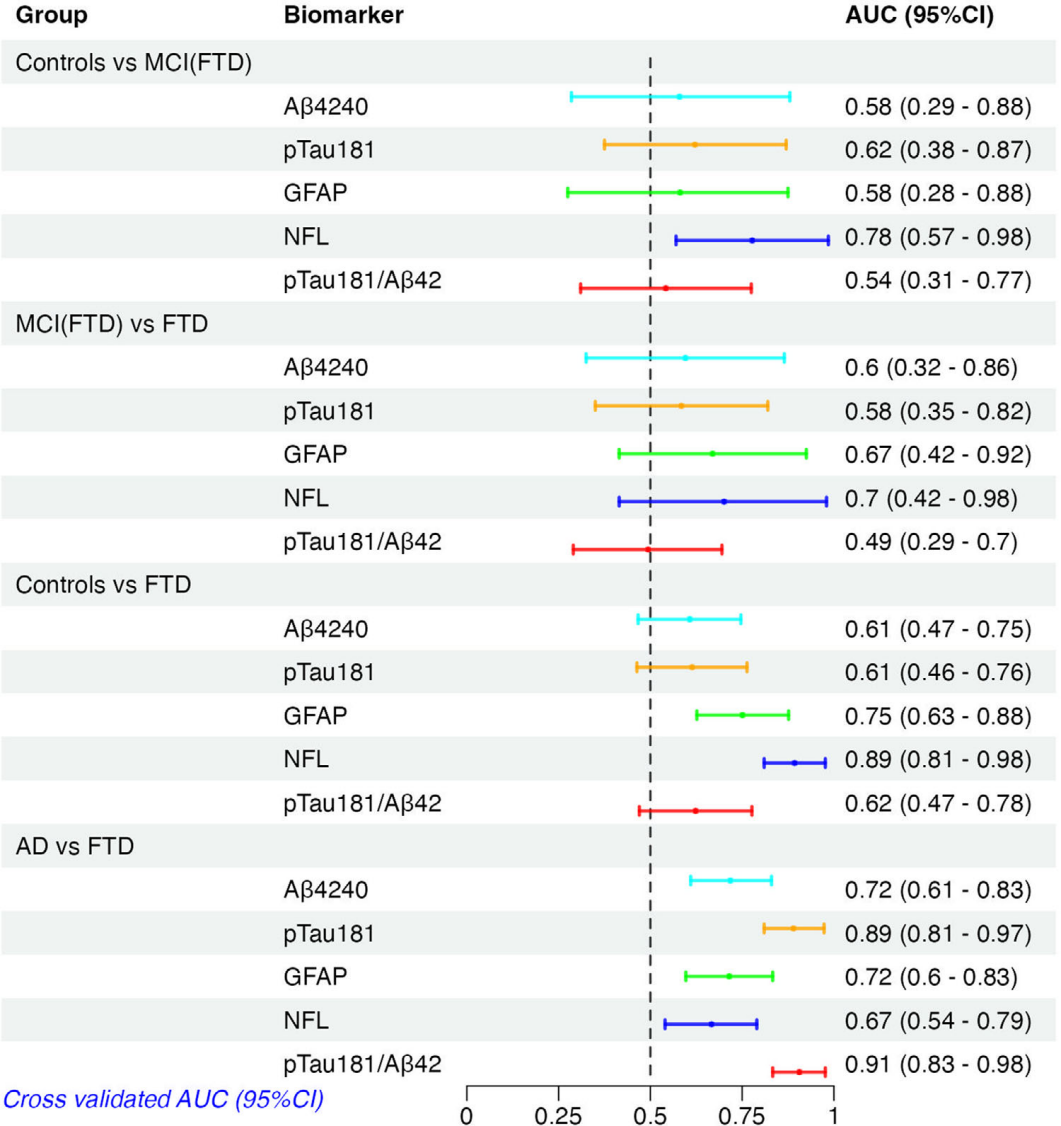
Internal cross‐validation AUC (95% CI) values for the FTD continuum and for FTD versus AD. Aβ, amyloid beta; AD, Alzheimer's disease; AUC, area under the curve; CI, confidence interval; FTD, frontotemporal dementia; GFAP, glial fibrillary acidic protein; NfL, neurofilament light chain; p‐tau, phosphorylated tau

Plasma NfL showed the strongest stepwise increase along the FTD continuum (Figure 1D). The levels of NfL in the MCI(FTD) group were as high as those observed in AD or DLB. Concerning AUC values, NfL had the only AUC value with the 95% CI not to cross 0.5 (AUC: 0.78 [95% CI: 0.57–0.99], Figure 4) to separate controls from MCI(FTD), while there were no biomarkers that could demarcate MCI(FTD) from FTD. The AUC for NfL was 0.7 indicating it was increased in FTD compared to MCI(FTD); however, given sample size, the 95% CI crossed 0.5. For the controls versus dementia (FTD) comparison, NfL had the highest AUC value (AUC: 0.89 [95% CI: 0.81–0.98]), and GFAP was the only other marker which showed significant discriminatory performance between the two groups (AUC: 0.75 [95% CI: 0.63–0.88]).

### 3.6 AD versus non‐AD dementias

Comparing AD to both DLB and FTD combined (non‐AD dementia group), p‐tau181 and the p‐tau181/Aβ42 ratio had the highest AUC values (Figure 2, 0.87 and 0.90, respectively), while all other markers and the multivariate set (Aβ40 × p‐tau181, and ratios Aβ42/GFAP and Aβ42/p‐tau181) had a maximum AUC of 0.72. Comparing AD versus FTD separately the p‐tau181/Aβ42 ratio was the best with an AUC of 0.91 (Figure 4). Comparing AD to DLB, the plasma p‐tau181/Aβ42 ratio was the marker that best discriminated these two groups with an AUC of 0.80, but this was strongly dependent upon the presence of Aβ co‐pathology. After stratification into with/without Aβ co‐pathology, the p‐tau181/Aβ42 ratio again was the top marker to discriminate AD from DLB without Aβ co‐pathology with an AUC of 0.90, while this decreased to 0.68 comparing AD versus DLB to Aβ co‐pathology (Figure 3). No other markers could discriminate AD from DLB–Aβ+ with all AUC values < 0.68 (Figure 3).

### 3.7 Cut‐off derivation via the dual cut‐off model for controls versus AD

**FIGURE 5:**
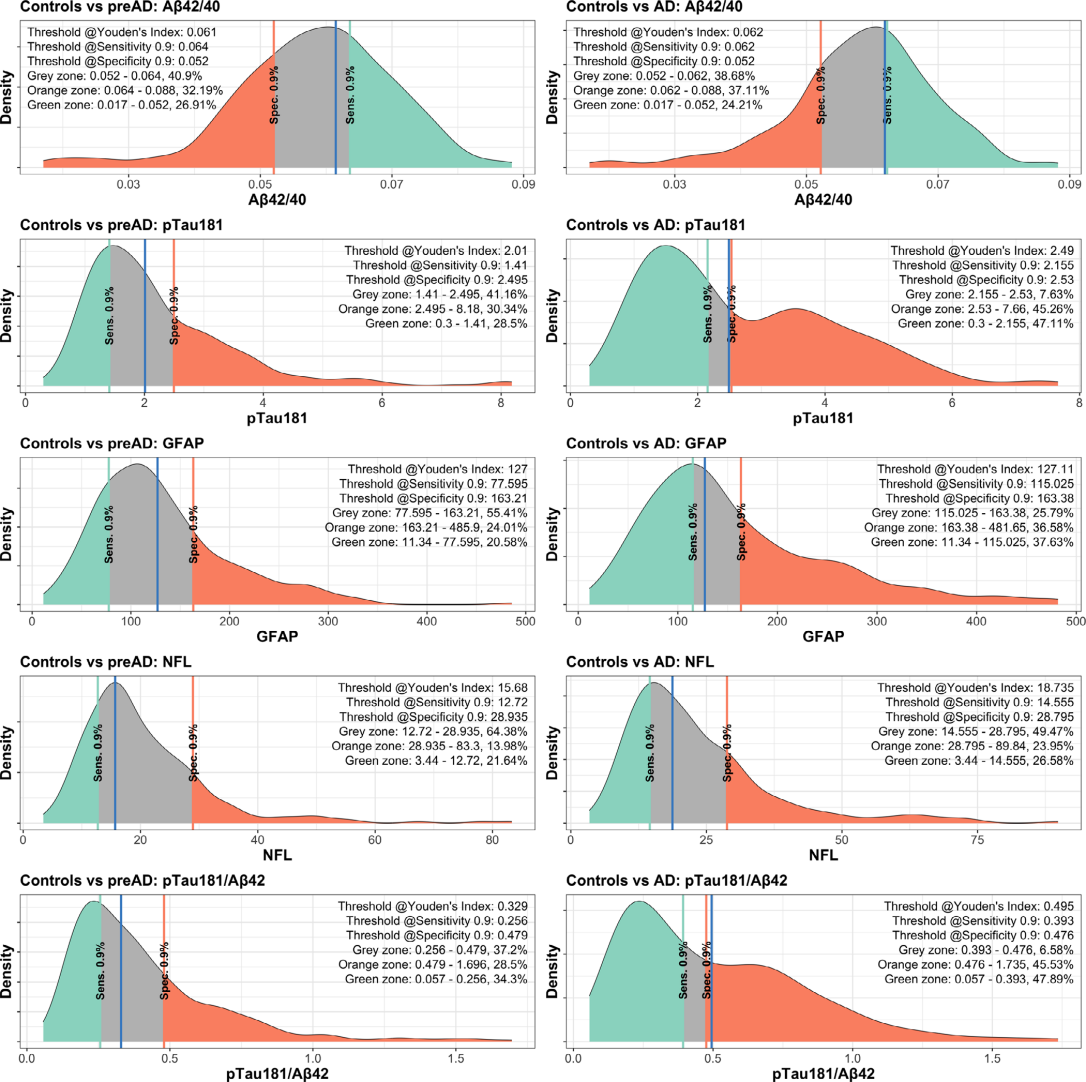
Biomarker cut‐offs for controls versus preAD and controls versus AD. Density plots for each biomarker with cut‐offs taken from the ROC calculated using controls versus AD. Percent of participants in gray zone calculated as the number of participants whose plasma biomarker concentration falls within the gray zone divided by the total number of participants. Blue line represents Youden index. Values shown for Aβ42, GFAP, NfL, and p‐tau181 are in pg/mL. Aβ, amyloid beta; AD, Alzheimer's disease; GFAP, glial fibrillary acidic protein; NfL, neurofilament light chain; preAD, pre‐clinical Alzheimer's disease; p‐tau, phosphorylated tau; Sens., sensitivity; Spec., specificity.

Separate to the single cut‐off results presented above, we next established dual cut‐off distribution plots deriving biomarker cut‐offs for 90% sensitivity and 90% specificity, allowing the determination of the gray zone (Figure 5; gray zone defines the proportion of participants between the two cut‐offs). Across the five main plasma biomarkers investigated, the marker showing the smallest gray zone was the p‐tau181/Aβ42 ratio (6.58%) and p‐tau181 (7.63%), followed by GFAP (25.79%), Aβ42/40 (38.68%), and NfL (49.47%). Translating this into the percentage of data rather than participants with values within the gray zone, we saw percentages of 4.95% for p‐tau181/Aβ42, 5.1% for p‐tau181, 10.28% for GFAP, 14.17% for the Aβ42/40 ratio, and 16.48% for NfL.

### 3.8 External cross‐validations of the cut‐offs

Figure S2 in supporting information shows a forest plot for AUC values from the cross‐validations (validation Method 1) of individual plasma biomarkers to discriminate dementia disease stage (not enough data for DLB) from controls using independent data from UNIPG (ALFA+ and BIODEGMAR were reserved for validation Method 2 only). In this cohort, p‐tau181 and the p‐tau181/Aβ42 ratio had AUC values ≈ 10% less at the preclinical stages (AUCs: p‐tau181: 0.73 [0.54–0.92], p‐tau181/Aβ42: 0.74 [0.54–0.93]) than in the multi‐center bPRIDE cohort (Figure 2), while at the prodromal stage, AUC values for p‐tau181 alone but not the ratio were similar (AUCs: p‐tau181: 0.88 [0.78–0.99], p‐tau181/Aβ42: 0.84 [0.68–0.99]). Despite p‐tau181 having similar performance as observed in bPRIDE at the AD dementia stage (AUC p‐tau181 validation: 0.93 [0.85–1.00], bPRIDE: 0.95 [0.90–1.00]), plasma GFAP was the best biomarker discriminating any stage of the AD continuum compared to controls and comparing AD to FTD in the validation cohort 1 (AUC: 0.96 [0.9–1.00], Figure S2).

We also investigated whether the cut‐offs derived from the multi‐center bPRIDE cohort (Table S3 in supporting information) could be applied to the independent cohorts (validation Method 2, UNIPG, ALFA+, and BIODEGMAR; Table S4 in supporting information), using both the Youden index calculated from bPRIDE and for 90% sensitivity or 90% specificity. Demographic characteristics, and study‐specific AUC values to predict disease group for each validation cohort are provided in Tables S5 and S6 in supporting information. AUC values for each comparison group were lower using validation Method 2 (fixed cut‐off method) compared to validation Method 1 (standard model training and test on separate data), suggesting slightly different distributions for each biomarker per cohort. We tested this in Figure S3A–C in supporting information, plotting the distributions for each biomarker for UNIPG, ALFA+, and BIODEGMAR cohorts. Plots include the original Youden index calculated within each study (black dotted line), along with the Youden's index from bPRIDE (blue line), and the cut‐offs at 90% sensitivity (green) and 90% specificity (orange). bPRIDE cut‐offs to separate disease status were different than cohort‐ specific cut‐offs for p‐tau181, GFAP, and NfL, but closer for Aβ42/40 (UNIPG only) across disease comparisons and validation cohorts.

## 4 DISCUSSION

In the present study we investigated the ability of plasma Aβ42/40, p‐tau181, p‐tau181/Aβ42, GFAP, and NfL to discriminate groups along the continuum of three neurological diseases: AD, FTD, and DLB, in a large international multi‐center cohort. Across the AD continuum, p‐tau181 had the best AUC values to separate all groups. Early in the AD continuum (preAD vs. controls), the multivariate model, including the interaction between Aβ40 and p‐tau181, and ratios Aβ42/GFAP and Aβ42/p‐tau181, produced slightly higher (≈ 5%) AUC values compared to individual markers, while this difference was reduced to 1% at the AD dementia stage compared to controls. Both p‐tau181 and the p‐tau181/Aβ42 ratio performed well to discriminate between AD and other dementias, except for DLB cases with amyloid co‐pathology. A dual cut‐off model defined a small gray zone containing a small number of participants for p‐tau181 and the p‐tau181/Aβ4 ratio (i.e., 7.6% of participants for p‐tau181 and 6.6% of participants for the p‐tau181/Aβ42 ratio).

The consistent increase in plasma p‐tau181 levels along the full AD continuum is in line with previous work,^45^, ^46^, ^47^ which reported increased p‐tau181 levels along the symptomatic stages of AD. Other p‐tau isoforms (e.g., p‐tau231, p‐tau217) have also shown similar behaviors, though p‐tau217 and p‐tau231 have demonstrated larger increases along AD stages. The increase of p‐tau181 in the preAD group suggests that it reflects tauopathy secondary to the presence of Aβ pathology at this early stage. The relationship between p‐tau181 and Aβ pathology is further supported by the evidence of increased p‐tau181 levels in DLB cases with Aβ co‐pathology. The stepwise increase in plasma p‐tau181 across the disease continuum may represent the combination of Aβ together with increasing tau tangle burden throughout the MCI and clinical AD stages.

Identification of a cheap and non‐invasive blood‐based biomarker is important for all three neurodegenerative disorders assessed here (AD, FTD, and DLB). While p‐tau181 and its ratio with Aβ42 were the best markers to detect AD along its continuum, NfL and GFAP had stronger performance to identify DLB and FTD, specifically where there was no amyloid co‐pathology. Both p‐tau181 and the p‐tau181/Aβ42 ratio had high AUC values to demarcate AD from both FTD and DLB, but only in those cases without amyloid co‐pathology. No biomarkers were, however, able to separate AD from DLB with amyloid co‐pathology, highlighting the need to identify additional biomarkers to separate these neurological conditions. These results are particularly relevant when considering preclinical stages of these diseases, when the presence of molecular pathology cannot be defined by other clinical measures. Furthermore, the results could have important implications for the timely inclusion of patients into clinical trials, especially those that aim to initiate treatment strategies to prevent the development of symptoms.

Using the dual cut‐off plots we found a small area of uncertainty (gray zone) for p‐tau181 compared to other biomarkers in identifying AD. Using information from the consensus statement from Schindler et al.,^48^ patients identified within the gray zone from either a confirmatory blood‐based‐biomarker (BBM, sensitivity and specificity ≥ 90%) or as a triaging test (sensitivity ≥ 90% and specificity ≥ 85%) should be referred to either PET or CSF Aβ testing to confirm amyloid pathology. The method was also useful to compare the biomarker densities and Youden's index between the bPRIDE study and validation studies. Plots demonstrated that differences in the underlying distribution of controls versus dementia impact the diagnostic cut‐offs, and small changes in these cut‐offs can impact the ability for external cross‐validation. Differences in pre‐analytical handling,^49^ fasting or non‐fasting,^50^ or prevalence of other confounding factors may be responsible for these small differences in cut‐off values, which are often seen between research studies. bPRIDE as a combination of six international studies provided cut‐offs that represent an average when using a large, combined cohort all assessed in one laboratory.

A further point to take into consideration when translating cut‐offs across cohorts is the fact that there can be slight differences in assay results due to kit–lot differences. Preliminary assessment of biomarkers prior to cross‐validations (controls vs. AD) showed variable but not significantly different AUC values across centers within bPRIDE. As such, comparing cut‐off values between bPRIDE and external cohorts when different kits–lots were used, we would expect to see differences in predictive accuracy. Given the lot‐to‐lot bridging could not be performed on the validation cohorts, it is possible that the validation AUC values are underestimated, representing a limitation of this work. Thus, while an international uniform cut‐off is still elusive, and may not be possible based on these biomarkers, cohort‐specific cut‐off values are best used for the most accurate diagnostic assessment. Use of dual cut‐off values for either high sensitivity or high specificity though are useful at least within the research setting to either rule out or rule in study participants for clinical trial suitability, and as such these methods provide useful information to reduce screening costs.

The present research has used well‐characterized cohorts from large international studies to compare plasma biomarker levels. While the overall sample size is high, the individual sub‐group analyses between some disease groups were relatively low, especially MCI(FTD) and MCI(DLB), and as such disease predictions need to be taken with caution. One limitation of this work was the sample size of the validation groups. Initial validation using the traditional train and test method on the UNIPG data set demonstrated high AUC values (Figure S2); however, upon further testing via the derived cut‐offs (bPRIDE) these values were lower (Table S3). Furthermore, upon testing validation cohorts ALFA+ and BIODEGMAR using the bPRIDE‐derived cut‐offs, AUC values were again reduced suggesting pre‐analytical factors and possibly machine settings at different locations may still play a role in biomarker distributions. While the performance of biomarkers in distinguishing groups along the AD, FTD, and DLB continuums has demonstrated moderate to high accuracies in our study, it is important to note that the study cohorts predominantly consisted of individuals of European or Australian descent. There was minimal representation from Asian, African American, or Latino populations. Consequently, further research is warranted to evaluate both the accuracy of these biomarkers in detecting diseases, and to establish assay‐specific cut‐off values, which may differ among diverse ethnic populations. Last, this project did not include other biomarkers which have shown strong accuracy in detecting AD pathology (p‐tau217: Simoa/Lumipulse/mass spectrometry, p‐tau231: Simoa),^39^, ^45^, ^51^, ^52^ and as such the comparative accuracy demonstrated here is limited to only the Simoa N4PE (Quanterix) and the Quanterix pTau181 assays. This work, however, did demonstrate a strong AUC value for controls versus AD (AUC: 0.95), similar to that seen for p‐tau217 to separate Aβ– from Aβ+ groups.^44^

This study represents a large collaborative effort to demonstrate the predictive capability of the plasma biomarkers Aβ42/40, GFAP, NfL, and p‐tau181. A multivariate model to predict Aβ pathology early in the AD continuum proved to be useful; however, at the later stage, using p‐tau181 alone was sufficient. Internal cross‐validations demonstrated good AUC values providing evidence that these markers are stable to be measured across different centers of inclusion around the world; however, external cross‐validations demonstrated difficulty in replicating predictive accuracy, providing evidence that more work is needed to address inter‐center differences in plasma biomarker levels.

## CONFLICT OF INTEREST STATEMENT

J.D., E.J.M., P.O.R., A.F.L., J.C., A.G.E., C.M., Y.P., A.T., M.K.S., I.H., L.B., C.F., K.V., S.I.V., and S.H. have no conflicts of interest to declare. G.B. has grants or contracts from the Parkinson's Foundation, Fujirebio, and the Alzheimer's Association. L.V. has grants or contracts from NWO VENI and Amsterdam UMC, ZonMw, Olink Dioraphte, Roche Diagnostics, Ely Lilly, Alzheimer's Association, Alzheimer Nederland, and Dutch Dementia researchers conference committee. D.A. has support from the Instituto de Salud Carlos III, and the Department of Health Generalitat de Catalunya PERIS program, and funding from Grfols S.A., Lily, Fujirebio, Roche Diagnostics, Nutricia, Krka Farmaceutical SL, Zambon SAU, Esteve Pharmaceuticals, and Neuraxpharm. N.M.C. received support from the Swedish Alzheimer Foundation, Family Ronnstrom Foundation, Swedish Brain Foundation, Kock Foundation, WASP and DDLS joint call for research projects, Konug Gustaf V:s och Drottning Victorias Frimurarestiftelse, the Swedish Research Council, Biogen, and Owkin. I.V. has grants, funding, or contracts from the Amsterdam UMC starter grant, and the TKI grant Health‐Holland, Neurogen Biomarking, and Quanterix. L.G. has received grants or contracts from AirAlzh Foundation; received consulting fees from Fujirebio and Eli Lilly; has payment or honoraria from Fujirebio, Eli Lilly, and Eisai; and support for attending meetings and/or travel from Fujirebio, Eli Lilly, and Novartis. L.G. also participates on a data safety monitoring board or advisory board for Eli Lilly. A.W. received support from Quanterix and a grant from the European Union's Horizon 2020 research and innovation program under the Marie Skłodowska‐ Curie. J.F. received support from Fondo de Investigaciones Sanitario (FIS); Instituto de Salud Carlos III, Spain; National Institutes of Health (NIH), USA; Generalitat de Catalunya, Spain; Fundació Tatiana Pérez de Guzmán el Bueno, Spain; Alzheimer ´s Association, USA; Brightfocus, USA; and Horizon 2020 (European Commission). J.F. received consulting fees from Lundbeck, Ionis, and AC Immune, and payments or honoraria from Roche Diagnostics, Esteve, Biogen, Laboratorios Canot, MKI, Eisai, Lilly, and Adamed. J.F. has patent issued; WO2019175379 A1 Markers of synaptopathy in neurodegenerative disease. J.F. participates on a data safety monitoring board or advisory board for AC Immune, Alzheon, Zambon, Lilly, Roche Diagnostics, Eisai, and Perha. J.F. has leadership or fiduciary roles in the Spanish Neurological Society, T21 Research Society, Lumind Foundation, Jerome‐Lejeune Foundation, Alzheimer's Association, Health Research Board, Dementia Trials Ireland, European Commission, National Institutes of Health, USA, and the Instituto de Salud Carlos III, Spain. J.F. has receipt of equipment, materials, drugs, medical writing, gifts. or other services from Life Molecular Imaging (LMI). M.O. received grants or contracts from the BMBF – FTLD consortium, moodmarker, the ALS association, and EU_MIRIADE, and funding from Biogen, Axon, Roche Diagnostics, and Grifols. M.O. participates on a data safety monitory or advisory board from the Biogen ATLAS trial, and has a leadership or fiduciary role in the German Society for CSF diagnostics and neurochemistry, as a speaker for the FTLD consortium, and for the Society for CSF diagnostics and neurochemistry. A.L. has grants or contracts from Hersenstichting and ZOnMW, and has a leadership or fiduciary role on the Steering Committee E‐DLB and the Dutch Neurology Society. W.F. has grants or contracts from ZonMW, NWO, EU‐FP7, EU‐JPND, Alzheimer Nederland, Hersenstichting CardioVascular Onderzoek Nederland, Health∼Holland, Topsector Life Sciences & Health, stichting Dioraphte, Gieskes‐Strijbis fonds, stichting Equilibrio, Noaber Foundation, Edwin Bouw fonds, Pasman stichting, Stichting Steun Alzheimercentrum Amsterdam, Philips, Biogen MA Inc, Novartis‐NL, Life‐MI, AVID, Roche BV, Fujifilm, Eisai, Combinostics. W.F. holds the Pasman chair. W.F. is recipient of ABOARD, which is a public–private partnership receiving funding from ZonMW (#73305095007) and Health∼Holland, Topsector Life Sciences & Health (PPP‐allowance; #LSHM20106). W.F. is recipient of TAP‐dementia, ZonMw #10510032120003. W.F. is recipient of IHI‐AD‐RIDDLE (#101132933), a project supported by the Innovative Health Initiative Joint Undertaking (IHI JU). W.F. is consultant to Oxford Health Policy, Forum CIC, Roche Diagnostics, Eisai, and Biogen MA Inc. W.F. has been an invited speaker at Boehringer Ingelheim, Biogen MA Inc, Danone, Eisai, WebMD Neurology (Medscape), NovoNordisk, Springer Healthcare, European Brain Council. W.F. participated on advisory boards of Biogen MA Inc, Roche Diagnostics, and Eli Lilly. WF is member of the steering committee of Novonordisk's Evoke/Evoke+ phase 3 trials. W.F. is member of the steering committee of PAVE, and Think Brain Health. W.F. was associate editor of *Alzheimer, Research & Therapy* in 2020/2021. W.F. is associate editor at *Brain*. J.H. has funding from SAB Eli Lilly and has stock in Alzheom company. O.H. has consulting fees from AC, Immune, BioArctic, Biogen, Bristol, Meyer, Squibb, C2N Diagnostics institute, Eisai, Eli Lilly, Fujirebio, Merck, Novartis, Novo, Nordisk, Roche Diagnostics, Sanofi, and Siemens. L.P. has funding from JPco‐fuND‐2: Multinational research projects on Personalised Medicine for Neurodegenerative Diseases (CUP number J99C18000210005). L.P. has grants or contracts European Union—Next Generation EU – PNRR M6C2 ‐ Investimento 2.1 Valorizzazione e potenziamento della ricerca biomedica del SSN (PNRR‐MAD‐2022‐12376035) and Fujirebio. A.L.B. had support from Fondo de Investigaciones Sanitario (FIS), Institution de Salud Carlos III AC19/00103; has grants or contracts from CIBERNED program (Program 1, Alzheimer Disease); has received consulting fees for Grifols S.A. and Lilly; and has patents planned issued or pending; WO2019175379 A1 Markers of synaptopathy in neurodegenerative disease. M.S.C. has grants or contracts from Roche Diagnostics, consulting fees from Roche Diagnostics, and has received funding from Roche, Almirall, Eli Lilly, and Novo Nordisk. M.S.C. has participated on data safety monitoring board or advisory boards for Roche Diagnostics, Grifols, and Eli Lilly. M.S.C. has received equipment, materials, drugs, medical writing gifts, or other services from Roche Diagnostics, Avid Radiopharmaceuticals, Inc. (Eli Lilly and company), Janssen Research and Development, ADx Neurosciences, Alamar Biosciences, Fujirebio, Meso Scale Discovery, and ALZpath. M.S.C. has other financial or non‐financial interests with Roche Diagnostics. A.P.P. has support from Laboratoris Esteve, Nutricia Ltd, and participates on a data safety monitory board or advisory board for Schwabe Farma Iberica. C.M. has grants or contracts from EuFingers JPND research grant, and the ADDF digital biomarkers research grant. M.d.C. has support from the JPND‐Bpride project funding, and grants or contracts from Alzheimer's research and therapy, Attraction Talent Comunidad de Madrid, and PROYECTOS I+D+I – 2020″‐ Retos de investigación from the Ministerio Español de Ciencia e innovación; has received payment or honoraria from Novonordisk and Springer Healthcare; and has a leadership or fiduciary role in BBB‐PIA chair AA and is Scientific advisor for ADPD. C.E.T. has research contracts with Acumen, ADx Neurosciences, AC‐Immune, Alamar, Aribio, Axon Neurosciences, Beckman‐Coulter, BioConnect, Bioorchestra, Brainstorm Therapeutics, Celgene, Cognition Therapeutics, EIP Pharma, Eisai, Eli Lilly, Fujirebio, Instant Nano Biosensors, Novo Nordisk, Olink, PeopleBio, Quanterix, Roche, Toyama, Vivoryon. She is editor in chief of *Alzheimer Research and Therapy*, and serves on editorial boards of *Molecular Neurodegeneratoin, Neurology: Neuroimmunology & Neuroinflammation, Medidact Neurologie*/Springer, and serves on committee to define guidelines for Cognitive disturbances, and one for acute Neurology in the Netherlands. She had consultancy/speaker contracts for Aribio, Biogen, Beckman‐Coulter, Cognition Therapeutics, Eli Lilly, Merck, Novo Nordisk, Olink, Roche, and Veravas. Author disclosures are available in the supporting information.

## CONSENT STATEMENT

All participants gave their written informed consent for use of their biological material and clinical data for research purposes. Ethical approval was granted by the ethical committee of each participating center.

## Supporting information



Supporting Information

Supporting Information
