# Supplementary material for: Diagnostic performance of plasma Aβ42/40 ratio, p‐tau181, GFAP, and NfL along the continuum of Alzheimer's disease and non‐AD dementias: An international multi‐center study
Source: Alzheimers Dement. 2025 Jun 23;21(6):e14573. doi: 10.1002/alz.14573 (PMC12185245; doi:10.1002/alz.14573)
Supplement: Supplementary file 1 — Supporting Information [file ALZ-21-e14573-s001.docx]

Supplementary section

Supplementary table 1: Study sample demographic characteristics and disease classifications

|  |  | Study Centre | | | | | |
| --- | --- | --- | --- | --- | --- | --- | --- |
|  | Total | AIBL | SPIN | UU | UNIPG | BioFINDER | ADC |
| Total N | 1287 | 203 | 241 | 197 | 200 | 205 | 241 |
| Mean age | 70.12 (7.14) | 71.92 (6.89) | 72.24 (7.29) | 68.05 (7.98) | 70.76 (6.75) | 71.35 (5.29) | 66.6 (6.43) |
| Gender (M%) | 622 (48%) | 96 (47%) | 123 (51%) | 104 (53%) | 83 (42%) | 59 (29%) | 157 (65%) |
| MMSE (median, MAD) | 26 (4.45) | 27 (2.97) | 25 (4.45) | 25 (4.45) | 25 (4.45) | 29 (1.48) | 25 (4.45) |
| Years of education, (median, SD) | 11 (4.45) | 12 (4.45) | 10 (2.97) | 12 (2.97) | 8 (4.45) | 12 (4.45) | 10 (2.97) |
| Controls | 198 | 26 | 20 | 13 | 40 | 78 | 21 |
| sMCI | 158 | 50 | 20 | 23 | 33 | 11 | 21 |
| preAD | 181 | 51 | 11 | 4 | 12 | 86 | 17 |
| MCI(AD) | 156 | 39 | 20 | 24 | 21 | 30 | 22 |
| AD | 182 | 37 | 23 | 49 | 52 | 0 | 21 |
| MCI(FTD) | 46 | 0 | 7 | 4 | 30 | 0 | 5 |
| FTD | 170 | 0 | 58 | 68 | 6 | 0 | 38 |
| MCI(DLB) | 25 | 0 | 15 | 0 | 3 | 0 | 7 |
| DLB (Aβ unknown) | 12 | 0 | 12 | 0 | 0 | 0 | 0 |
| DLB (Aβ-) | 60 | 0 | 17 | 11 | 0 | 0 | 32 |
| DLB (Aβ+) | 99 | 0 | 38 | 1 | 3 | 0 | 57 |

preAD: pre-clinical AD; AD: Alzheimer’s disease; MCI: Mild Cognitive Impairment; DLB: Dementia with Lewy Bodies; FTD: Frontotemporal Dementia. AIBL: Australian Imaging, Biomarkers and Lifestyle; SPIN: Sant Pau Initiative on Neurodegeneration, UU: Ulm University; UNIPG: University of Perugia; ADC: Amsterdam Dementia Cohort; Aβ: amyloid Beta; M: Male; MAD: median absolute deviation; SD: standard deviation.

Supplementary table 2: Initial assessment of predictive performance across centre

| **Biomarker** | **Validation using cohort** | **AUC (95%CI)** | **AUC range** | **Sensitivity** | **Specificity** | **PPV** | **NPV** | **Accuracy** |
| --- | --- | --- | --- | --- | --- | --- | --- | --- |
| pTau181 | AUS | 0.975 (0.95 - 1) | 0.068 | 91.89 | 96.15 | 97.14 | 89.29 | 93.65 |
|  | BCN | 0.954 (0.9 - 1) |  | 86.96 | 90 | 90.91 | 85.71 | 88.37 |
|  | DE | 0.953 (0.9 - 1) |  | 79.59 | 100 | 100 | 56.52 | 83.87 |
|  | IT | 0.956 (0.92 - 0.99) |  | 84.62 | 90 | 91.67 | 81.82 | 86.96 |
|  | VUmc | 0.907 (0.81 - 1) |  | 76.19 | 95.24 | 94.12 | 80 | 85.71 |
|  | bPRIDE (overall)* | 0.949 (0.90 - 1) |  | 83.85 | 94.28 | 94.77 | 78.67 | 87.71 |
|  | bPRIDE (overall) & | 0.946 (0.92 - 0.97) |  | 84.07 | 89.9 | 88.44 | 85.99 | 87.11 |
|  | bPRIDE (overall) % | 0.955 (0.94 - 0.97)^ |  | 85.16 | 92.42 | 91.18 | 87.14 | 88.95 |
| Aβ42/40 | AUS | 0.82 (0.69 - 0.95) | 0.157 | 81.08 | 80.77 | 85.71 | 75 | 80.95 |
|  | BCN | 0.933 (0.86 - 1) |  | 95.65 | 80 | 84.62 | 94.12 | 88.37 |
|  | DE | 0.851 (0.72 - 0.99) |  | 79.59 | 84.62 | 95.12 | 52.38 | 80.65 |
|  | IT | 0.781 (0.69 - 0.88) |  | 59.62 | 90 | 88.57 | 63.16 | 72.83 |
|  | VUmc | 0.776 (0.61 - 0.94) |  | 95.24 | 71.43 | 76.92 | 93.75 | 83.33 |
|  | bPRIDE (overall)* | 0.832 (0.71 - 0.95) |  | 82.24 | 81.36 | 86.19 | 75.68 | 81.23 |
|  | bPRIDE (overall) & | 0.805 (0.76 - 0.85) |  | 90.66 | 61.62 | 69.57 | 85.33 | 75.79 |
|  | bPRIDE (overall) % | 0.921 (0.89 - 0.95)^ |  | 90.66 | 78.28 | 79.33 | 90.12 | 84.21 |
| GFAP | AUS | 0.89 (0.81 - 0.97) | 0.049 | 89.19 | 73.08 | 82.5 | 82.61 | 82.54 |
|  | BCN | 0.925 (0.85 - 1) |  | 86.96 | 85 | 86.96 | 85 | 86.05 |
|  | DE | 0.912 (0.81 - 1) |  | 87.76 | 84.62 | 95.56 | 64.71 | 87.1 |
|  | IT | 0.939 (0.9 - 0.98) |  | 84.62 | 87.5 | 89.8 | 81.4 | 85.87 |
|  | VUmc | 0.923 (0.85 - 1) |  | 100 | 66.67 | 75 | 100 | 83.33 |
|  | bPRIDE (overall)* | 0.918 (0.84 - 0.99) |  | 89.71 | 79.37 | 85.96 | 82.74 | 84.98 |
|  | bPRIDE (overall) & | 0.878 (0.84 - 0.91) |  | 85.16 | 75.76 | 76.35 | 84.75 | 80.26 |
|  | bPRIDE (overall) % | 0.885 (0.85 - 0.92) |  | 77.27 | 0.885 | 77.72 | 85.96 | 81.58 |
| NFL | AUS | 0.833 (0.73 - 0.93) | 0.098 | 83.78 | 69.23 | 79.49 | 75 | 77.78 |
|  | BCN | 0.83 (0.71 - 0.95) |  | 82.61 | 70 | 76 | 77.78 | 76.74 |
|  | DE | 0.738 (0.59 - 0.89) |  | 89.8 | 46.15 | 86.27 | 54.55 | 80.65 |
|  | IT | 0.819 (0.73 - 0.91) |  | 86.54 | 70 | 78.95 | 80 | 79.35 |
|  | VUmc | 0.836 (0.71 - 0.96) |  | 85.71 | 71.43 | 75 | 83.33 | 78.57 |
|  | bPRIDE (overall)* | 0.811 (0.69 - 0.93) |  | 85.69 | 65.36 | 79.14 | 74.13 | 78.62 |
|  | bPRIDE (overall) & | 0.781 (0.74 - 0.83) |  | 76.37 | 67.68 | 69.15 | 75.98 | 72.37 |
|  | bPRIDE (overall) % | 0.885 (0.85 - 0.92)^ |  | 87.36 | 76.26 | 77.18 | 86.78 | 81.58 |
| pTau181/Aβ42 | AUS | 0.983 (0.96 - 1) | 0.074 | 100 | 92.31 | 94.87 | 100 | 96.83 |
|  | BCN | 0.957 (0.91 - 1) |  | 95.65 | 80 | 84.62 | 94.12 | 88.37 |
|  | DE | 0.992 (0.98 - 1) |  | 95.92 | 100 | 100 | 86.67 | 96.77 |
|  | IT | 0.967 (0.94 - 1) |  | 80.77 | 100 | 100 | 80 | 89.13 |
|  | VUmc | 0.918 (0.82 - 1) |  | 85.71 | 90.48 | 90 | 86.36 | 88.1 |
|  | bPRIDE (overall)* | 0.963 (0.92 - 1) |  | 91.61 | 92.56 | 93.9 | 89.43 | 91.84 |
|  | bPRIDE (overall) & | 0.937 (0.91 - 0.96) |  | 82.97 | 92.93 | 91.52 | 85.58 | 88.16 |
|  | bPRIDE (overall) % | 0.955 (0.94 - 0.97)^ |  | 93.41 | 86.36 | 86.29 | 93.44 | 89.74 |
| Multivariate | AUS | 0.952 (0.88 - 1) | 0.084 | 100 | 92.31 | 94.87 | 100 | 96.83 |
|  | BCN | 0.985 (0.96 - 1) |  | 95.65 | 95 | 95.65 | 95 | 95.35 |
|  | DE | 1 (1 - 1) |  | 100 | 100 | 100 | 100 | 100 |
|  | IT | 0.979 (0.96 - 1) |  | 96.15 | 90 | 92.59 | 94.74 | 93.48 |
|  | VUmc | 0.916 (0.83 - 1) |  | 80.95 | 90.48 | 89.47 | 82.61 | 85.71 |
|  | bPRIDE (overall)* | 0.966 (0.93 - 1) |  | 94.55 | 93.56 | 94.52 | 94.47 | 94.27 |
|  | bPRIDE (overall) & | 0.957 (0.94 - 0.98) |  | 95.05 | 83.33 | 83.98 | 94.83 | 88.95 |
|  | bPRIDE (overall) % | 0.959 (0.94 - 0.98) |  | 86.81 | 92.42 | 91.33 | 88.41 | 89.74 |

Analyses to test assay performance between sites only performed for the control’s vs AD comparison.

* bPRIDE (overall): All ROC statistics calculated using the rolling circle cross validation technique

& bPRIDE (overall): All ROC statistics calculated using the complete data set without cross validation

% bPRIDE (overall): All ROC statistics calculated using the complete data set without cross validation adjusted for centre using first three principal components

^ bPRIDE (overall) not cross validated and adjusting for three principal components significantly different to bPRIDE (overall) not cross validated.

pTau181, GFAP, NFL and the pTau181/Aβ42 ratio AUC values not significantly different across all centres. AB42/40 AUC value nominally significantly different between BCN & IT, p=0.014, BCN vs VUmc p=0.095, however neither retained significance post adjustment for multiple comparisons.

Supplementary Table 3: Biomarker cut-offs per disease group

|  |  | Cut-offs | | |
| --- | --- | --- | --- | --- |
| Group comparison | Biomarker | @Youden's Index | @90% Sensitivity | @90% Specificity |
| Controls vs preAD | Aβ42 | 6.155 | 7.085 | 4.095 |
|  | Aβ42/40 | 0.0615 | 0.064 | 0.052 |
|  | pTau181 | 2.01 | 1.41 | 2.495 |
|  | GFAP | 127 | 77.595 | 163.21 |
|  | NFL | 15.68 | 12.72 | 28.935 |
| Controls vs MCI(AD) | Aβ42 | 5.05 | 7.27 | 4.095 |
|  | Aβ42/40 | 0.0571 | 0.063 | 0.052 |
|  | pTau181 | 2.315 | 1.715 | 2.535 |
|  | GFAP | 145.995 | 86.155 | 162.54 |
|  | NFL | 18.75 | 13.225 | 28.88 |
| Controls vs AD | Aβ42 | 5.815 | 6.995 | 4.095 |
|  | Aβ42/40 | 0.0619 | 0.062 | 0.052 |
|  | pTau181 | 2.49 | 2.155 | 2.53 |
|  | GFAP | 127.11 | 115.025 | 163.38 |
|  | NFL | 18.735 | 14.555 | 28.795 |
| Controls vs DLB | Aβ42 | 6.015 | 8.385 | 4.82 |
|  | Aβ42/40 | 0.0664 | 0.072 | 0.054 |
|  | pTau181 | 1.514 | 0.965 | 2.14 |
|  | GFAP | 123.67 | 43.97 | 123.67 |
|  | NFL | 14.475 | 8.875 | 20.11 |
| Controls vs FTD | Aβ42 | 6.54 | 4.28 | 8.185 |
|  | Aβ42/40 | 0.0563 | 0.075 | 0.052 |
|  | pTau181 | 1.405 | 0.955 | 2.545 |
|  | GFAP | 149.66 | 61.09 | 163.735 |
|  | NFL | 18.71 | 16.55 | 28.935 |
| AD vs FTD | Aβ42 | 6.52 | 6.985 | 4.06 |
|  | Aβ42/40 | 0.0623 | 0.062 | 0.049 |
|  | pTau181 | 2.855 | 2.15 | 3.11 |
|  | GFAP | 126.23 | 114.39 | 240.39 |
|  | NFL | 33.5 | 48.94 | 16.655 |

preAD: pre-clinical AD; AD: Alzheimer’s disease; MCI: Mild Cognitive Impairment; DLB: Dementia with Lewy Bodies; FTD: Frontotemporal Dementia. Cut-off values shown for Aβ42, GFAP, NFL and pTau181 are in pg/mL.

Supplementary Table 4: Biomarker cross validations using pre-determined cut-offs

|  |  | AUC (95%CI) | | |
| --- | --- | --- | --- | --- |
| Group | Biomarker | UNIPG*^ι^ | ALFA+^ι^ | BIODEGMAR^ι^ |
| Controls vs preAD | Aβ42/40 | 0.83 (0.69 - 0.97) | 0.6 (0.55 - 0.64) | 0.65 (0.59 - 0.71) |
|  | pTau181 | 0.6 (0.41 - 0.78) | 0.64 (0.59 - 0.69) | 0.71 (0.64 - 0.78) |
|  | GFAP | 0.73 (0.57 - 0.9) | 0.64 (0.59 - 0.69) | 0.74 (0.67 - 0.81) |
|  | NFL | 0.67 (0.49 - 0.84) | 0.57 (0.53 - 0.62) | 0.57 (0.52 - 0.62) |
| Controls vs MCI(AD) | Aβ42/40 | 0.61 (0.46 - 0.75) |  | 0.73 (0.66 - 0.8) |
|  | pTau181 | 0.79 (0.66 - 0.93) |  | 0.74 (0.66 - 0.81) |
|  | GFAP | 0.77 (0.63 - 0.91) |  | 0.75 (0.68 - 0.82) |
|  | NFL | 0.68 (0.53 - 0.83) |  | 0.58 (0.52 - 0.64) |
| Controls vs AD | Aβ42/40 | 0.76 (0.6 - 0.92) |  | 0.65 (0.59 - 0.71) |
|  | pTau181 | 0.86 (0.72 - 0.99) |  | 0.72 (0.64 - 0.79) |
|  | GFAP | 0.86 (0.73 - 0.98) |  | 0.74 (0.67 - 0.81) |
|  | NFL | 0.82 (0.68 - 0.96) |  | 0.58 (0.52 - 0.64) |

preAD: pre-clinical AD; AD: Alzheimer’s disease; MCI: Mild Cognitive Impairment; AUC: Area Under the Curve; CI: Confidence Interval. * comparison group are participants with Subjective Cognitive Decline, not controls. ^ι^ AUC values with 95%CI created using binary biomarkers with bPRIDE-defined cut-offs vs disease outcome.

Supplementary Table 5: Validation cohort demographic details

| Characteristic | UNIPG | | | ALFA+ | |  | BIODEGMAR | | | |
| --- | --- | --- | --- | --- | --- | --- | --- | --- | --- | --- |
|  | All | Aβ- | Aβ+ | All | Aβ- | Aβ+ |  | All | Aβ- | Aβ+ |
| N | 74 | 15 | 59 | 400 | 265 | 135 | N | 173 | 61 | 112 |
| Mean age (SD) | 71.6 (7.3) | 64.8 (8.9) | 73.4 (5.5) | 61.1 (4.7) | 60.6 (4.5) | 62.2 (5.0) | Mean age (SD) | 73.8 (5.3) | 71.3 (6.5) | 75.1 (4.0) |
| Gender (M%) | 32 (43%) | 10 (63%) | 22 (38%) | 154 (38.5%) | 100 (37.7%) | 54 (40%) | Gender (M%) | 64 (37.0%) | 26 (42.6%) | 38 (33.9%) |
| Controls | 15 | 15 | 0 |  |  |  |  |  |  |  |
| preAD | 16 | 0 | 16 | 400 | 265 | 135 | Controls* (CDR=0) | 7 (4.0%) | 6 (9.8%) | 1 (0.9%) |
| MCI(AD) | 28 | 0 | 28 | 0 | 0 | 0 | MCI (CDR=0.5) | 56 (32.4%) | 25 (41.0%) | 31 (27.7%) |
| AD | 15 | 0 | 15 | 0 | 0 | 0 | Dementia (CDR>=1) | 110(63.6%) | 30 (49.2%) | 80 (71.4%) |

*Controls within the BIODEGMAR cohort are participants with subjective cognitive decline. Aβ: amyloid Beta; N: Number; M: Male; preAD: pre-clinical AD; MCI: Mild cognitive impairment; AD: Alzheimer’s disease; SD: Standard deviation; CDR: Clinical dementia rating

Supplementary Table 6: Validation cohort AUC and biomarker cut-offs

| Characteristic | UNIPG (controls vs AD) | | ALFA+ (Controls vs pre-AD) | | BIODEGMAR (non-AD vs AD CSF profile) | |
| --- | --- | --- | --- | --- | --- | --- |
|  | AUC (95%CI) | Youden's Index | AUC (95%CI) | Youden's Index | AUC (95%CI) | Youden's Index |
| Aβ42/40 | 0.78 (0.59 – 0.97) | 0.06 | 0.75 (0.70 – 0.80) | 0.072 | 0.74 (0.65-0.82) | 0.0571 |
| pTau181 | 0.93 (0.85 – 1) | 2.952 | 0.68 (0.62 – 0.73) | 2.167 | 0.77 (0.69-0.86) | 2.317 |
| GFAP | 0.96 (0.90 – 1) | 171.201 | 0.68 (0.63 – 0.74) | 121.998 | 0.81 (0.74-0.88) | 141.703 |
| NFL | 0.90 (0.77 – 1) | 21.786 | 0.63 (0.58 – 0.69) | 12.389 | 0.62 (0.53-0.72) | 20.710 |

AUC: Area Under the Curve; CI: Confidence interval; preAD: pre-clinical AD; MCI: Mild cognitive impairment; AD: Alzheimer’s disease; CSF: Cerebrospinal fluid. Cut-off values at Youden’s Index shown for Aβ42, GFAP, NFL and pTau181 are in pg/mL.

Supplementary Figure 1: Principal component analyses of all markers from all centres.


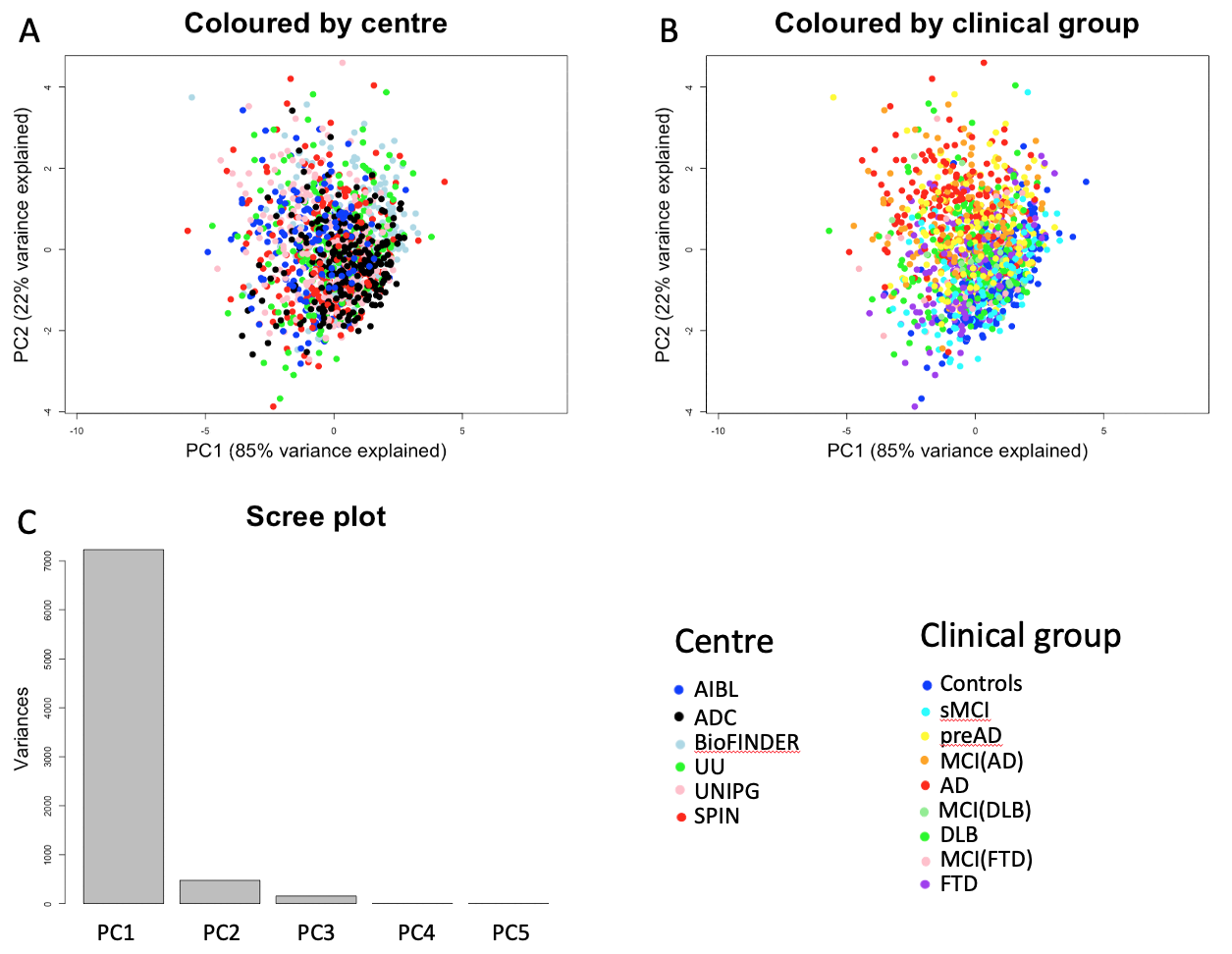


Principal component analyses of plasma biomarker concentrations from all participants. A) Coloured by centre. B) Coloured by clinical group. C) Scree plot of variance explained per principal component.

Supplementary Figure 2: External cross validations (UNIPG) of AUC values for the prediction of neurological disease from controls participants.


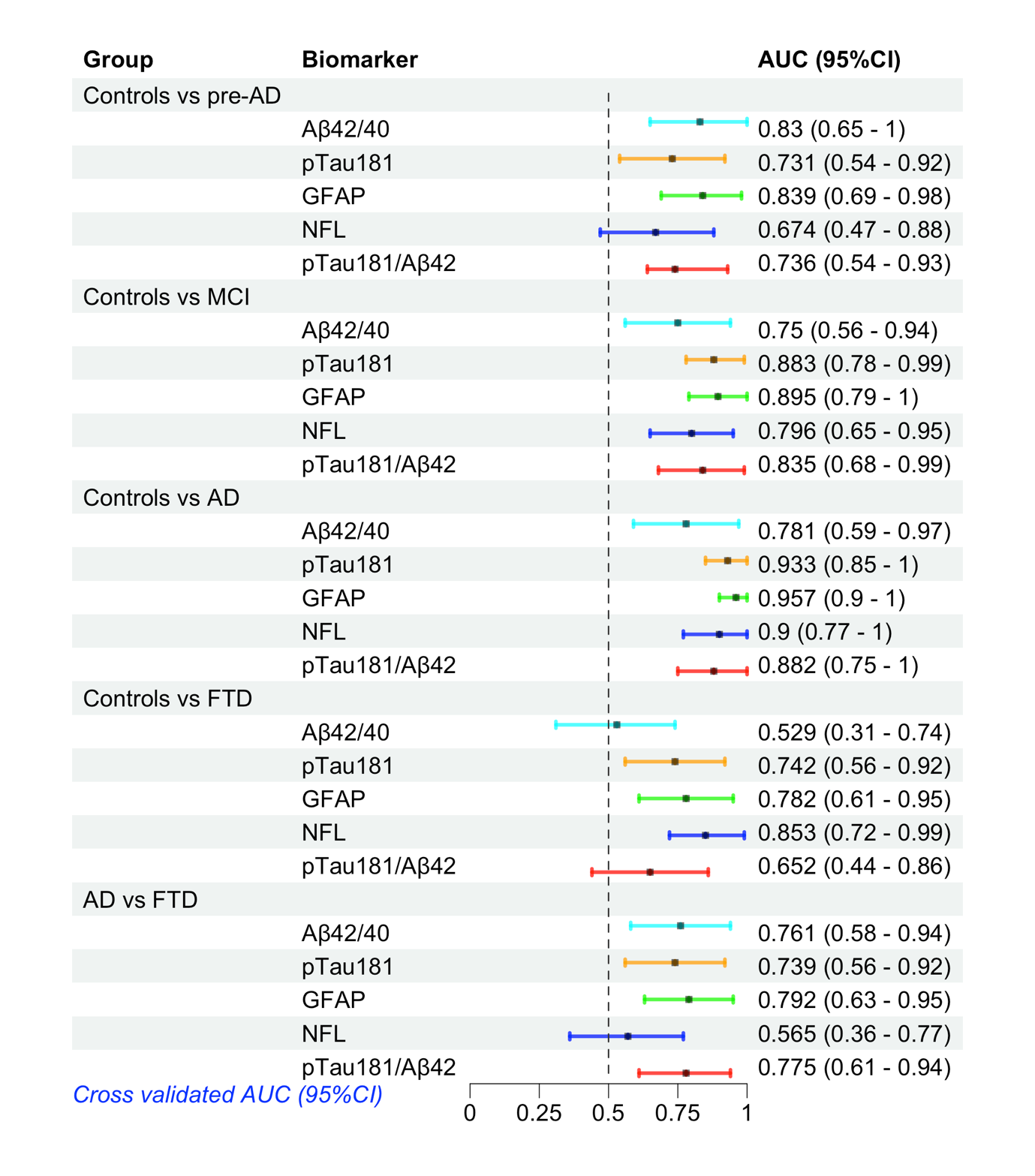


preAD: pre-clinical AD; AD: Alzheimer’s disease; MCI: Mild Cognitive Impairment; FTD: Frontotemporal Dementia; AUC: Area Under the Curve; AUC: Area Under the Curve; CI: Confidence Interval.

Supplementary Figure 3A: Density plots for UNIPG


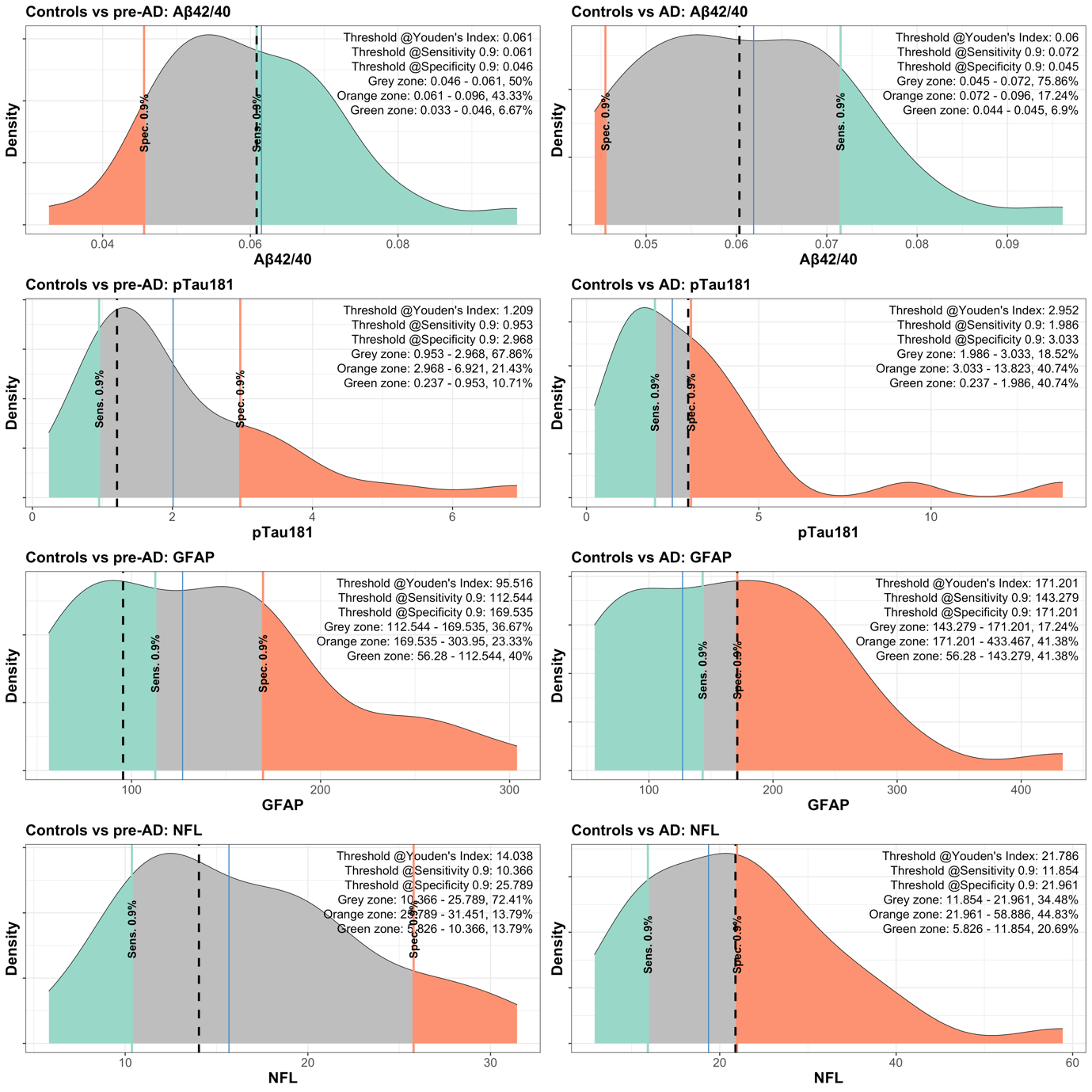


Density plots for each biomarker with cut-offs taken from the ROC calculated using Controls vs pre-AD (left) and Controls vs AD (right). Percent of participants in each zone calculated as the number of participants whose plasma biomarker concentration falls within each zone divided by the total number of participants. Study original Youden’s Index calculated within each study shown as black dotted line. Youden’s Index from bPRIDE shown as solid blue line. Sens.: Sensitivity; Spec.: Specificity; AD: Alzheimer’s disease. Values shown for Aβ42, GFAP, NFL and pTau181 are in pg/mL.

Supplementary Figure 3B: Density plots for ALFA+

Density plots for each biomarker with cut-offs taken from the ROC calculated using Controls vs pre-AD. Grey zone calculation: absolute difference in biomarker values between cut-offs at 90% Sensitivity and 90% Specificity divided by the range for each biomarker (maximum – minimum). Percent of participants in each zone calculated as the number of participants whose plasma biomarker concentration falls within each zone divided by the total number of participants. Study original Youden’s Index calculated within each study shown as black dotted line. Youden’s Index from bPRIDE shown as solid blue line. Sens.: Sensitivity; Spec.: Specificity; AD: Alzheimer’s disease. Values shown for Aβ42, GFAP, NFL and pTau181 are in pg/mL.

Supplementary Figure 3C: Density plots for BIODEGMAR

Density plots for each biomarker with cut-offs taken from the ROC calculated using non-ADF CSF vs AD CSF profile Percent of participants in each zone calculated as the number of participants whose plasma biomarker concentration falls within each zone divided by the total number of participants. Study original Youden’s Index calculated within each study shown as black dotted line. Youden’s Index from bPRIDE shown as solid blue line. CSF: Cerebrospinal fluid; Sens.: Sensitivity; Spec.: Specificity; AD: Alzheimer’s disease. Values shown for Aβ42, GFAP, NFL and pTau181 are in pg/mL.
